# Identification of the Complex Interplay Between Nematode-Related lncRNAs and Their Target Genes in *Glycine max* L.

**DOI:** 10.3389/fpls.2021.779597

**Published:** 2021-12-10

**Authors:** Masoumeh Ahmadi Khoei, Marzieh Karimi, Roya Karamian, Sahand Amini, Aboozar Soorni

**Affiliations:** ^1^Department of Biology, Faculty of Sciences, Bu-Ali Sina University, Hamedan, Iran; ^2^Independent Researcher, Isfahan, Iran; ^3^Independent Researcher, Tabriz, Iran; ^4^Department of Biotechnology, College of Agriculture, Isfahan University of Technology, Isfahan, Iran

**Keywords:** nematode, soybean, long non-coding RNA, regulation, plant defense, bioinformatics

## Abstract

Soybean (*Glycine max*) is a major plant protein source and oilseed crop. However, plant-parasitic nematodes (PPNs) affect its annual yield. In the current study, in order to better understand the regulation of defense mechanism against PPNs in soybean, we investigated the role of long non-coding RNAs (lncRNAs) in response to two nematode species, *Heterodera glycines* (SCN: soybean cyst nematode) and *Rotylenchulus reniformis* (reniform). To this end, two publicly available RNA-seq data sets (SCN data set and RAD: reniform-associated data set) were employed to discover the lncRNAome profile of soybean under SCN and reniform infection, respectively. Upon identification of unannotated transcripts in these data sets, a seven-step pipeline was utilized to sieve these transcripts, which ended up in 384 and 283 potential lncRNAs in SCN data set and RAD, respectively. These transcripts were then used to predict cis and trans nematode-related targets in soybean genome. Computational prediction of target genes function, some of which were also among differentially expressed genes, revealed the involvement of putative nematode-responsive genes as well as enrichment of multiple stress responses in both data sets. Finally, 15 and six lncRNAs were proposed to be involved in microRNA-mediated regulation of gene expression in soybean in response to SNC and reniform infection, respectively. Collectively, this study provides a novel insight into the signaling and regulatory network of soybean-pathogen interactions and opens a new window for further research.

## Introduction

Soybean [*Glycine max* (L.) Merrill.], belonging to the Fabaceae family, is a highly nutritious leguminous crop (Massey et al., 2001; Alghamdi et al., 2018; Espina et al., 2018) and a high-quality protein source in human diet and livestock feed due to its well-balanced essential amino acids (Singer et al., 2019) Furthermore, soybean, by making up around 59% of the overall world oilseed production, is also a major oilseed crop (El-Hamidi and Zaher, 2018). In recent years, soybean oil has been employed as an industrial ingredient for the production of plastics, inks, papers, pesticides, varnishes, pharmaceutical compounds, and cosmetics (Alghamdi et al., 2018). Besides, soybean oil is a promising source of biodiesel fuel to exploit as an alternative biofuel and renewable energy to decelerate toxic greenhouse gas emissions currently, the United States, Brazil, Argentina, China, India, Paraguay, and Canada are leading producers of this commercially important crop in the world with an estimated annual production of ~341.1 million metric tonnes in 2020.^1^ However, multiple (a) biotic factors, including infectious disease agents, pests, and weeds, as well as extreme heat and cold, high salinity, drought, and waterlogging (Tran and Mochida, 2010; Bandara et al., 2020), cause a large reduction in quality and productivity, as well as a serious economic loss in soybean crop. Among limiting factors, PPNs are one of the most important agents that significantly affect soybean performance and yield by 10–15% (Lima et al., 2018).

In the midst of various nematode species associated with soybean, soybean cyst nematode (SCN; *Heterodera glycines*), reniform nematode (semi-endoparasitic nematode; *Rotylenchulus reniformis*), root-knot nematode (*Meloidogyne incognita*), and lesion nematode (*Pratylenchus brachyurus*) are the most deleterious pests (Lima et al., 2017; Klepadlo et al., 2018). For instance, 3–4 generations of SCN can reportedly parasitize soybean in a single growing season (Neupane et al., 2019a). Nowadays, nematicide and nematode-resistant cultivars, rotating to non-host crops, and biological controls are the most common strategies to control nematode infection and limit its impact on soybean productivity (Engelbrecht et al., 2020). However, it has been demonstrated that nematodes can develop very intricate parasitizing mechanisms to neutralize resistance strategies and establish the specific nematode feeding sites (NFSs) in host root cells *via* penetration into them and induction of the morphological, biochemical, and molecular change (Ali et al., 2017). Indeed, nematode secretions contain effector proteins that can modulate plant gene signaling cascades to induce NFSs and circumvent the host’s defense responses (Gheysen and Mitchum, 2011; Hewezi, 2015). Therefore, comprehensive studies are required to neutralize the ruinous effects of nematodes and dissect the underlying resistance mechanism of soybean as a host plant. Until now, several studies, especially the ones based on comparative transcriptome analysis of resistant and susceptible soybean genotypes, have been accomplished on the nematode-soybean interactions and the complex molecular mechanisms in response to nematode infestation (Klink and Matthews, 2009; Rambani et al., 2015; Tian et al., 2017; Neupane et al., 2019a). These studies have led to identification and characterization of various key genes involved in plant perception systems for parasite recognition, phytohormone-mediated defense responses, the mitogen-activated protein kinase (MAPK) signaling cascade, and WRKY-involved regulation (Beneventi et al., 2013; Wan et al., 2015; Yang et al., 2017; Zhang et al., 2017; Guo et al., 2019).

Despite these findings, additional studies are needed to explore and confer the resistance mechanisms to nematodes in soybean plant. One of the complementary analyses that could be applied to explore the potential pathways and novel components involved in plant-nematode interaction is the characterization and functional annotation analyses of the long non-coding RNAs (lncRNAs). LncRNAs are ≥200-nucleotide (nt) regulators that have multiple mechanisms of action *via* epigenetic, transcriptional, and post-transcriptional regulation of gene expression in response to various stress. For example, modulating the transcriptional machinery of plants has been proved by several lncRNAs. For instance, the regulatory role of two lncRNAs COOLAIR and COLDAIR on flowering locus C (*FLC*) expression (Heo and Sung, 2011), the regulatory role of lncRNA SVALKA on C-repeat/dehydration responsive element binding factor 1 (*CBF1*) under cold stress (Kindgren et al., 2018), and the regulatory role of *ELENA1* on pathogenesis-related 1 (*PR1*) gene against bacterial pathogens (Seo et al., 2017) have provided the compelling evidences of gene regulation by lncRNAs. LncRNAs can mediate chromatin modifications and thus promote or repress gene expression. They can also have a cofactor role along with transcription factors and control gene expression (Cabili et al., 2011; Marchese et al., 2017). Furthermore, although called non-coding RNA, they are shown to code for proteins that can be involved in gene regulation. At the post-transcriptional level, lncRNAs can interfere with microRNAs (miRNAs) and neutralize their silencing role and thus upregulate the transcript level. For example, in *Brassica juncea*, the potential interaction between miR-172 and lncRNA TCONS_00047156 under heat stress, (Bhatia et al., 2020), in cassava, the potential interplay between linRNA159 and 340, as two cold stress-related lncRNAs, and miR-164 and miR-169, respectively (Li et al., 2017), and in tomato the interaction between lncRNA39026 and miR168a against *Phytophthora infestans* infection have been detected. Additionally, lncRNAs can recruit complementary small interfering RNAs (siRNAs) to regulate a gene expression (Wierzbicki, 2012; Böhmdorfer et al., 2014). Another mechanism is the regulation of expression of adjacent protein-coding genes in the genome, which is known as cis-regulation. Finally, lncRNAs can identify complementary mRNA sequences and interfere with the RNA editing and consequently with the expression of the paired gene (Kornienko et al., 2013; Zhao et al., 2019). In this regard, studying the structure and sequence of lncRNAs as well as prediction and functional enrichment analysis of their cis- and trans-target genes can be performed utilizing accurate computational tools (Johnson and Guigó, 2014; Wan et al., 2020; Fort et al., 2021). Besides, weighted gene co-expression network analyses (WGCNA) can be recruited to identify lncRNA-mRNA interactions. This analysis, as a momentous system biology-based approach, integrates independent large-scale gene expression profiling data sets into gene co-expression modules based on similarities in their expression profiles (Amrine et al., 2015; Li et al., 2018a). Since these modules are mostly enriched for genes that contributed to similar biological processes (BPs), it would be possible to predict putative functions of genes and decipher the novel genes and lncRNAs associated with the desired traits through modeling the lncRNA-mRNA co-expression network (Langfelder and Horvath, 2008; Serin et al., 2016; van Dam et al., 2018; Wang et al., 2019). Additionally, in each module, by measuring the intramodular connectivity, central players as hubs can be screened (Langfelder et al., 2013) and exploited as the candidate biomarkers.

Getting advantage of the high-throughput RNA-seq data, the involvement of lncRNAs has already been demonstrated in diverse and fundamental BPs (Shafiq et al., 2016; Singh et al., 2018). Similarly, several studies have explicated the lncRNA-mediated gene regulation in different plant-pathogen interactions (Li et al., 2018c,d; Yu et al., 2020; Zhang et al., 2020; Summanwar et al., 2021); however, only a few studies have investigated their regulatory roles in response to nematode invasion (Li et al., 2018a,b; Muthusamy et al., 2019). Since PPNs are one of the most threatening factors against soybean production, more in-depth studies are required to expand our knowledge on signaling events mediating the interplay between this crop and nematodes. Hence, in this study, two previously published RNA-seq data of nematode-infected soybean roots were employed to identify lncRNAs and conduct further analyses. Multi-filter lncRNA identification pipeline resulted in a total of 526 unique potential lncRNAs in two data sets. Various *in silico* analyses on these identified transcripts showed the extensive potential role of lncRNAs in nematode response *via* targeting multiple molecular pathways in soybean. Altogether, this research provided novel insights into the role of lncRNAs in biotic stress, as well as molecular signaling and regulation of nematode response in soybean.

## Materials and Methods

### Transcriptomic Data

In this study, two previously published transcriptome data sets, available in the European Nucleotide Archive (ENA, https://www.ebi.ac.uk/ena) under the BioProject accession numbers PRJNA306741 (Li et al., 2018b) and PRJNA348534 (Redding, 2018), were employed to identify nematode-related lncRNAs. In these experiments, cDNA libraries generated using NEBNext® Ultra™ RNA Library Prep Kit for Illumina® (NEB, United States) following the manufacturer’s instructions. Briefly, to construct libraries, first-strand cDNA was synthesized using mRNA purified from total RNA, random hexamer primers, and M-MuLV reverse transcriptase (RNase H-). Second-strand cDNA was synthetized using DNA polymerase I and RNase H. Next, after end repairing and 3′ end polyadenylation, NEBNext adaptor was ligated. PCR was performed using purified fragments with 150–200bp in length, universal PCR primers, and index (X) primer. Finally, double-end sequencing was performed by HiSeq instrument (Illumina, San Diego, United States). The first data set (SCN data set) contained 12 cDNA libraries representing the SCN-infected root samples of *Glycine max* Huipizhi Heidou, an SCN-resistant line (ZDD2315), at 5, 10, and 15 (N5, N10, and N15) days post-infection (dpi) and uninfected root samples at the same time points as the control in three biological replicates (Li et al., 2018b). The second data set (RAD: reniform-associated data set) consisted of 24 libraries, illustrating the infected (DAI3I, DAI6I, DAI9I, and DAI12I) and control (DAI3C, DAI6C, DAI9C, and DAI12C) root samples of *Glycine max* “Hutcheson” cultivar 3, 6, 9, and 12days after inoculation (DAI) with three biological replicates for each sample (Redding, 2018).

### Identification of Unannotated Transcripts and lncRNAs

**Figure 1:**
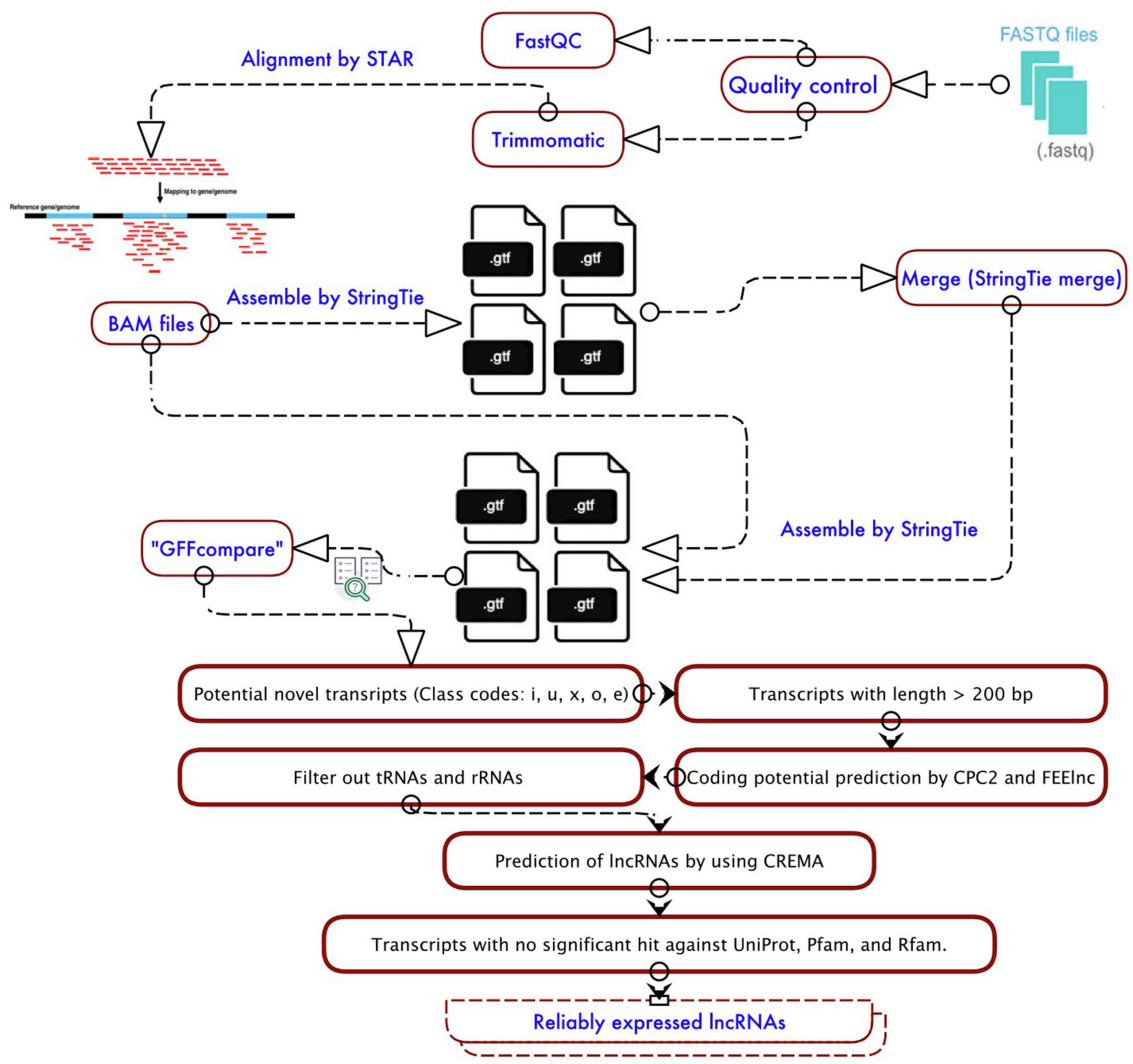
Workflow of lncRNA identification from RNA-Seq data sets.

According to the workflow shown in Figure 1, in the beginning, the selected data sets were individually analyzed by the Galaxy website (version 21.01; Jalili et al., 2021) for quality control, mapping, and identification of lncRNAs. The quality of sequences was assessed using FastQC (v.0.11.8; https://www.bioinformatics.babraham.ac.uk/projects/fastqc/), and then, low-quality bases were dropped using the Trimmomatic tool (v.0.38; Bolger et al., 2014). Trimmomatic parameters included trimming the bases with a quality score<Q20 and discarding reads with length<50bp. Then, high-quality clean reads were aligned to the reference genome of soybean (Wm82.a2.v1, https://soybase.org/GlycineBlastPages/blast_descriptions.php ) using STAR v.2.7.8a (Dobin and Gingeras, 2015). Transcripts aligned to the reference genome were assembled and quantified using StringTie v.2.1.1 (Pertea et al., 2015). The assembled transcripts were merged by StringTie’s merge tool to generate a consolidated annotation file to re-estimate transcript abundances.

To identify unannotated transcripts, transcriptome assemblies of each sample produced by StringTie were compared with the output of StringTie merge tool using gffcompare program. The output included the unannotated transcripts classified in different class codes of “u” (intergenic lncRNAs), “x” (antisense lncRNAs), “i” (intronic lncRNAs), “o” (generic exonic overlap lncRNAs with reference transcripts), and “e” (single exon transfrag overlapping a reference exon). Finally, using BEDTools v.2.29.2 (Quinlan and Hall, 2010), the names of unannotated transcripts were defined in a BED file and extracted from the soybean reference genome.

Since these identified unannotated transcripts may include potential coding genes, they were subjected to the following multi-step filtering approach:

1. The unannotated transcripts with more than 200 nucleotides and counts per million (CPM) >1 were extracted.
2. The output of the previous step was inputted into tRNAscan-SE 2.0 (Lowe and Chan, 2016) and then Barrnap 0.9^2^ to filter out possible transfer RNAs (tRNAs) and ribosomal RNAs (rRNAs).
3. Coding potential calculator (CPC2) software (Kang et al., 2017) and FEELnc v.0.2 (Wucher et al., 2017) with a shuffle mode (−m “shuffle”) were employed to evaluate the coding potential of predicted lncRNAs.
4. CREMA, available at www.github.com/gbgolding/crema (Simopoulos et al., 2018), was used to improve the specificity and accuracy of lncRNA prediction and ranking.
5. Ultimately, the transcripts with at least one significant (E-value, 1e-5) hit against the UniProt release 2021-02, Pfam release 34.0, and Rfam 14.5 database, which encoded a conserved protein/domain were excluded.

### Identification of Differentially Expressed mRNAs and lncRNAs

The differential expression analysis was conducted on the gene read count data matrices produced by python script prepDE.py. For this analysis, the generated matrices were uploaded onto the IDEAMEX website (Jimenez-Jacinto et al., 2019), and then, DESeq2 (Love et al., 2014) software was used to identify differentially expressed genes (DEGs) as well as the differentially expressed lncRNAs (DE-lncRNA) with screening parameters of FDR ≤0.05, log_2_ fold change (logFC) ≥2, and CPM=1.

### Identification of Monotonically Expressed lncRNAs

To identify monotonically expressed lncRNAs (ME-lncRNAs) whose expression patterns were highly correlated with the time series upon nematode infestation, MFSelector (monotonic feature selector) method (Wang et al., 2015) was applied to the SCN data set and RAD. The significance level of these patterns was evaluated utilizing a permutation test. Two distinct sets of ME-lncRNAs (with corresponding *p*-values) were found with ascending or descending monotonic expression patterns. To meet the efficient level of stringency for monotonicity, we defined the parameters as permut=100, svdetimes=100, and svdenoise=0.1, according to (Rajavel et al., 2021). Eventually, ME-lncRNAs with a CPM >1 and sample variance for discriminating error value ≤1 were considered as significant ME-lncRNAs.

### Functional Annotation and Enrichment Analysis

To get functional insights into identified lncRNAs, a series of computational approaches and tools were applied aiming at homology search and characterization of transposable element (TE) content of lncRNAs, functional enrichment analysis on the neighboring genes of lncRNAs, and prediction of interaction between DE-lncRNA and DEGs. Since many known lncRNAs have displayed sequence conservation among various plant species, the comparison between lncRNA sequences across different species can provide profound insights into the evolutionary conservation of lncRNAs. Hence, we compared the identified lncRNAs in this study with the lncRNAs available in CANTATAdb v.2.0 ( http://cantata.amu.edu.pl/ ; Szcześniak et al., 2019), Green Non-Coding Database (GREENC; http://greenc.sequentiabiotech.com/wiki/Main_Page ; Gallart et al., 2016), and Plant Long non-coding RNA Database (PLncDB v.2.0; http://plncdb.tobaccodb.org/ ; Jin et al., 2021), using the BLASTN tool with the criteria of e-value 1e5, identity >70%, and query coverage >30%. In further assessment, we compared the lncRNAs to those lncRNA sets introduced by Golicz et al. (2018) and Lin et al. (2020) to further validate the pulled-out lncRNAs (Golicz et al., 2018; Lin et al., 2020).

Besides, we compared the identified lncRNAs to the soybean TE database obtained from SoyBase (SoyBase_TE_Fasta.txt), using BLASTN with the same criteria noted above. Due to the cis-mode regulation of lncRNAs, they are capable of targeting the neighboring genes. Accordingly, in this study, the biological function of genes located 100 Kbp upstream and downstream of lncRNAs as cis-regulated potential target genes was investigated *via* Gene Ontology (GO) and Kyoto Encyclopedia of Genes and Genomes (KEGG) pathway enrichment analysis. Additionally, to unravel the interaction between lncRNA and DEGs, the trans-regulated target genes of lncRNA were predicted using LncTar (Li et al., 2014) with default parameters.

### Identification of lncRNAs as miRNA Endogenous Target Mimic

Owing to the presence of partial complementarity between lncRNAs and the miRNAs, lncRNAs can act as miRNA target mimics and negatively regulate and sequester the activity of the miRNAs. Regarding this ability, herein, utilizing all the identified lncRNAs of the SCN data set and RAD as well as soybean miRNAs downloaded from miRBase release 22.0, we predicted miRNA mimic sites using psMimic software (Wu et al., 2013). Furthermore, the plant miRNA target prediction software, psRobot toolbox ( http://omicslab.genetics.ac.cn/psRobot/ ; Wu et al., 2012), was employed to identify the putative target genes of the miRNAs that exhibited mimicry with lncRNAs. The following parameters were set for this analysis: penalty score threshold=2.5, 5′ boundary of essential sequence=2, 3′ boundary of essential sequence=17, the maximal number of permitted gaps=1, and position after which with gaps permitted=17.

### Plant Materials and Validation by Real-Time PCR

To confirm the expression levels of predicted lncRNAs, plant materials and nematodes were prepared according to Li et al. (2018b) for SCN and Redding (2018) for reniform. For this purpose, first, a combination of 1% sodium hypochlorite and 70% (v/v) ethyl alcohol (EtOH) was used for 3min to surface sterilize the seeds of the SCN-resistant *Glycine max* (Huipizhi Heidou) line ZDD2315 and reniform nematode-susceptible *Glycine max* cultivar “Hutcheson” (Redding et al., 2018; Li et al., 2018b). The sterilized seeds were then planted in pots filled with equal parts of sterilized soil and sand mixture and subsequently incubated in a growth chamber at 28±2°C and 50% relative humidity with 14/10-h (light/dark) photoperiod. Then, the SCN race 3 was isolated from soil, hatched from the eggs, and matured to second-stage juveniles (J2) according to (Li et al., 2018b) protocol and introduced to soybean roots (Li et al., 2018b). The SCN-infected and uninfected root samples (as control) were harvested at the three time points including, 5, 10, and 15 dpi in three biological replicates. The reniform nematode inoculums were extracted according to Redding’s (2018) protocol and introduced to soybean roots. The infected and the control root samples were collected 3, 6, 9, and 12 DAI in three biological replicates (Redding et al., 2018). Total RNA of root samples was extracted by the column RNA isolation kit (DENAzist Asia Co., Iran). The first-strand cDNA was synthesized using 1μg of total RNA per sample and random hexamer primers by RevertAid First Strand cDNA Synthesis Kit (Thermo Fisher Co., United States), according to the manufacturer’s instructions. Using SYBR Green PCR Master Mix (BioFACT, Korea) and specific primers (Supplementary Table S1), the Real-Time PCR (qPCR) reaction was performed in a final volume of 15μl on the ABI system with three technical replicates per each biological replicate (ABI ViiA 7 Real-time PCR). The qPCR program included a single step of initial denaturation at 95°C for 10s, followed by 40cycles of 95°C for 5s and 60°C for 20s. *EF1B* and *UKN2* (Ma et al., 2013) housekeeping genes were used as internal reference genes to normalize the expression data. Finally, the relative expression levels of lncRNAs were calculated using the 2^-ΔΔCt^ method (Livak and Schmittgen, 2001).

### Coding/Non-coding Gene Co-expression Study

The WGCNA (Langfelder and Horvath, 2008) R package was employed to identify the regulatory lncRNA-mRNA co-expression networks through unveiling similar expression patterns between lncRNAs and mRNAs in response to SCN and reniform nematode invasion. To run WGCNA analysis, the normalized fragments per kilobase of transcript per million fragments mapped (FPKM) values of DE-lncRNAs and DEGs were imported. Then, to generate a similarity matrix, Pearson’s correlation between the log_2_ (FPKM+1) values of all gene pairs was calculated. Next, the similarity matrix was transformed into an adjacency matrix. The soft threshold power (β) of 9 was determined based on the scale-free topology criterion (Zhang and Horvath, 2005). Afterward, the adjacency matrix was used to compute the topological overlap measure (TOM) and corresponding dissimilarity (1-TOM). Subsequently, the clusters of densely interconnected genes (modules) were detected using hierarchical clustering of 1-TOM and the DynamicTree Cut algorithm (Langfelder and Horvath, 2008). Besides, as the phenotypic trait, we calculated the correlations among gene expression modules and “days after nematode infection.”

## Results

### Identification and Characterization of lncRNAs

To identify nematode-related lncRNAs in soybean, we used two transcriptome data sets, namely, SCN data set and RAD. In the SCN data set, ~711.2 million raw reads resulting from the sequencing of 12 cDNA libraries were available. Since reads in each library exhibited high-quality bases, trimming operation was skipped, and they were directly mapped to the soybean reference genome. Following the mapping, on average, over 85% of reads were aligned to the genome. In RAD, a total of 619 million raw reads with an average of 25.8 million reads per sample were obtained from the sequencing of 24 libraries. After trimming low-quality bases (score<Q20), high-quality reads with a minimal length of 50bp were mapped to the soybean reference genome. The mapping rate of reads in each library ranged between 88 and 94%.

**Figure 2:**
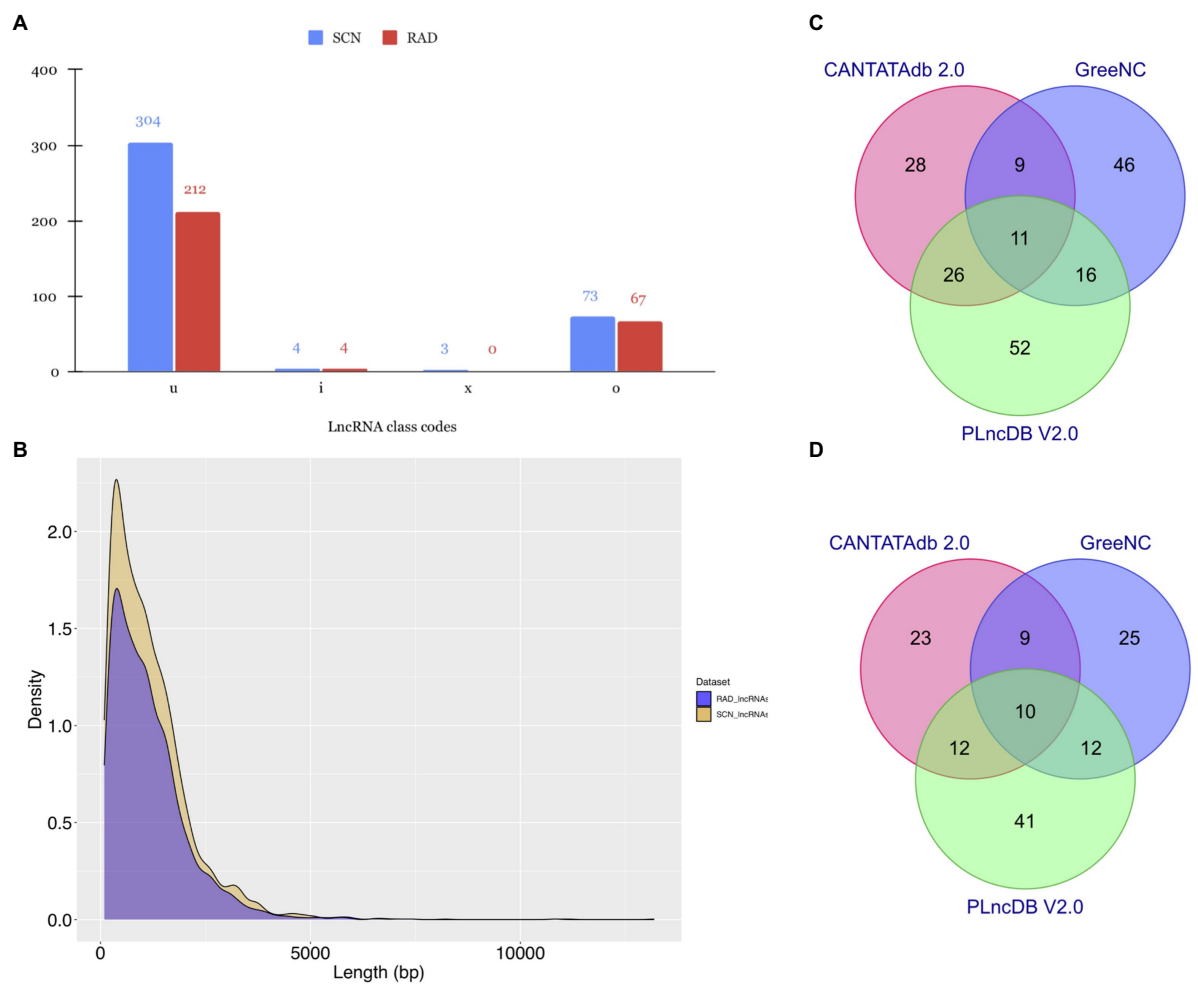
Characteristic features of lncRNAs identified in SCN data set and RAD. **(A)** Subdivision of lncRNAs according to the class codes (“u,” “x,” “i,” “o,” and “e”) determined by StringTie. **(B)** Length distribution of lncRNAs. Venn diagrams depicting the BLAST results of lncRNAs in **(C)** SCN data set and **(D)** RAD against CANTATAdb 2.0, GreeNC, and PLncDB v.2.0 databases.

After reconstructing the transcriptome for each sample and combining assemblies using StringTie (Pertea et al., 2015), a total of 106,215 and 119,916 transcripts were assembled in SCN data set and RAD, respectively. Of those, 2,275 (related to SCN data set) and 2,746 (related to RAD) transcripts were selected as unannotated transcripts (belonging to the class code of “u,” “x,” “i,” “o,” or “e”) and subjected to the lncRNA identification pipeline (Figure 2A). In the first step of lncRNA discovery, 1,331 and 1,359 unannotated transcripts were screened with CMP >1 in SCN data set and RAD, respectively. Out of these transcripts, respectively, 1,285 and 1,286 potential lncRNAs were identified using FEELnc software (Wucher et al., 2017). Subsequently, the potential lncRNAs were assessed to filter out the potential coding transcripts and possible rRNAs and tRNAs, remaining 791 and 801 transcripts in SCN data set and RAD, respectively. Next, using CREMA, respectively, 590 and 435 transcripts with a prediction score>0.5 were chosen as candidate lncRNAs. The last filtration was conducted to remove the transcripts homologous to the protein-coding genes and known protein domains documented in the Pfam database, as well as the transcripts homologous to the housekeeping RNAs (including tRNAs, rRNAs, snRNAs, and snoRNAs) in the Rfam database. This filtration resulted in 384 and 283 potential lncRNAs in SCN data set and RAD, respectively. The output of each filtering step in the lncRNA identification pipeline is presented in Table 1. Sequences of the identified lncRNAs, along with their genomic locations, are provided in Data S1.

**Table 1:** Performance of each filtering step in the lncRNA identification pipeline in SCN data set and RAD.

Step	SCN data set	RAD
Total transcripts assembled by StringTie	106,215	119,916
Potential novel transcripts (class code: i, u, x, o, e)	2,275	2,746
Filter transcripts with CPM >1	1,331	1,359
Coding potential prediction by FEElnc	1,285	1,286
Filter transcripts with length>200bp, and <10,000bp	1,174	1,194
Coding potential prediction by CPC2	804	814
Removal of rRNAs and tRNAs	791	801
Prediction of lncRNAs by CREMA	590	435
BLAST against UniProt, Pfam, and Rfam databases and removal of the significant hits	384	283
Potential lncRNAs with differential expression	51	111

As shown in Figure 2A, most of the identified lncRNAs in both data sets belonged to the “u” class code with 304 (79%) and 212 (74.9%) lncRNAs, respectively. The “o” class was the second dominated class, containing 73 (19%) and 67 (23.67%) lncRNAs in SCN data set and RAD, respectively. In both data sets, the least amount of lncRNAs fell into the “i” and “x” classes. Further investigation on the length distribution of lncRNAs indicated that the length density distribution of lncRNAs in SCN data set was not much different from those of RAD, and the majority of lncRNAs were shorter than 2,500nt (Figure 2B). However, in a specific length (200–7,967nt), SCN data set’s lncRNAs appeared in a higher density than the RAD’s lncRNAs (Figure 2B).

### Homology Search

To get a deeper understanding about the evolutionary conservation of lncRNAs, here we aligned all identified lncRNAs with previously predicted plant lncRNA sequences available in the CANTATAdb v.2.0, GreeNC, and PLncDB v.2.0. According to the BLAST search results, 188 SCN-related lncRNAs (~ 49% of all identified lncRNAs in the SCN data set) had at least one significant hit (Supplementary Table S2) in at least one of the non-coding databases, whereas, of 283 putative lncRNAs identified in the RAD, 132 lncRNAs (~ 47%) showed at least one significant hit (Supplementary Table S3). In detail, 7.29 and 8.13% of detected lncRNAs in SCN data set and RAD had significant hits only in the CANTATAdb database. Around 12% of lncRNAs in SCN data set and 8.83% in RAD showed significant homology with lncRNA sequences in the GreeNC database. In contrast, a higher proportion of lncRNAs, 13.54 and 14.49%, respectively, in SCN data set and RAD, had significant hits in PLncDB. Finally, only 2.86% of lncRNAs in SCN data set (Figure 2C) and 3.53% in RAD (Figure 2D) showed significant hits in all three databases.

To further verify the reliability of identified lncRNAs, we compared the pulled-out lncRNAs to those provided in soybean (Golicz et al., 2018; Lin et al., 2020). The results indicated that 81 lncRNAs found in the SCN data set had significant homology with 76 loci in the study of Golicz et al. (2018), while 236 lncRNAs were homologous to 229 loci detected by Lin et al. (2020) (Golicz et al., 2018; Lin et al., 2020). Among RAD lncRNAs, 51 lncRNAs were matched to 48 loci identified by Golicz et al. (2018), while 173 lncRNAs had homology with 166 loci in the study of Lin et al. (2020). In a further assessment using the expression profile data provided by Golicz et al. (2018), the lncRNAs that had significant homology with lncRNAs in Golicz’s study and were highly expressed in roots were identified and visualized *via* heatmaps (Supplementary Figures S1, S2). Their expression patterns in our data sets were also investigated. Among these identified lncRNAs, only two lncRNAs (MSTRG1600.1 and MSTRG1206.1) were found to be expressed in the SCN data set. The expression of MSTRG1600.1 was detected in infected samples so that the incremental changes in the expression of this lncRNA were observed from N5 toward N15. Likewise, an increased expression was detected for MSTRG1206.1 in N15 compared to N10. This remarkable upward expression trend of these two lncRNAs can reveal their potential role in soybean immune response to SCN. On the other hand, our results demonstrated the expression of two RAD lncRNAs (MSTRG.17157.1 and MSTRG.16268.1) in different control and infected samples. The remarkable upregulation of MSTRG.17157.1 was observed in the last time point after nematode inoculation (DAI12I) compared to DAI6I and DAI9I treatments. In contrast, MSTRG.16268.1 was only expressed in DAI9I and DAI3I, with a downregulation in DAI9I compared to DAI3I. Following these results, these two lncRNAs can be considered as potential lncRNAs responsive to the reniform nematode invasion.

### Characterization of TE Composition of lncRNAs

**Figure 3:**
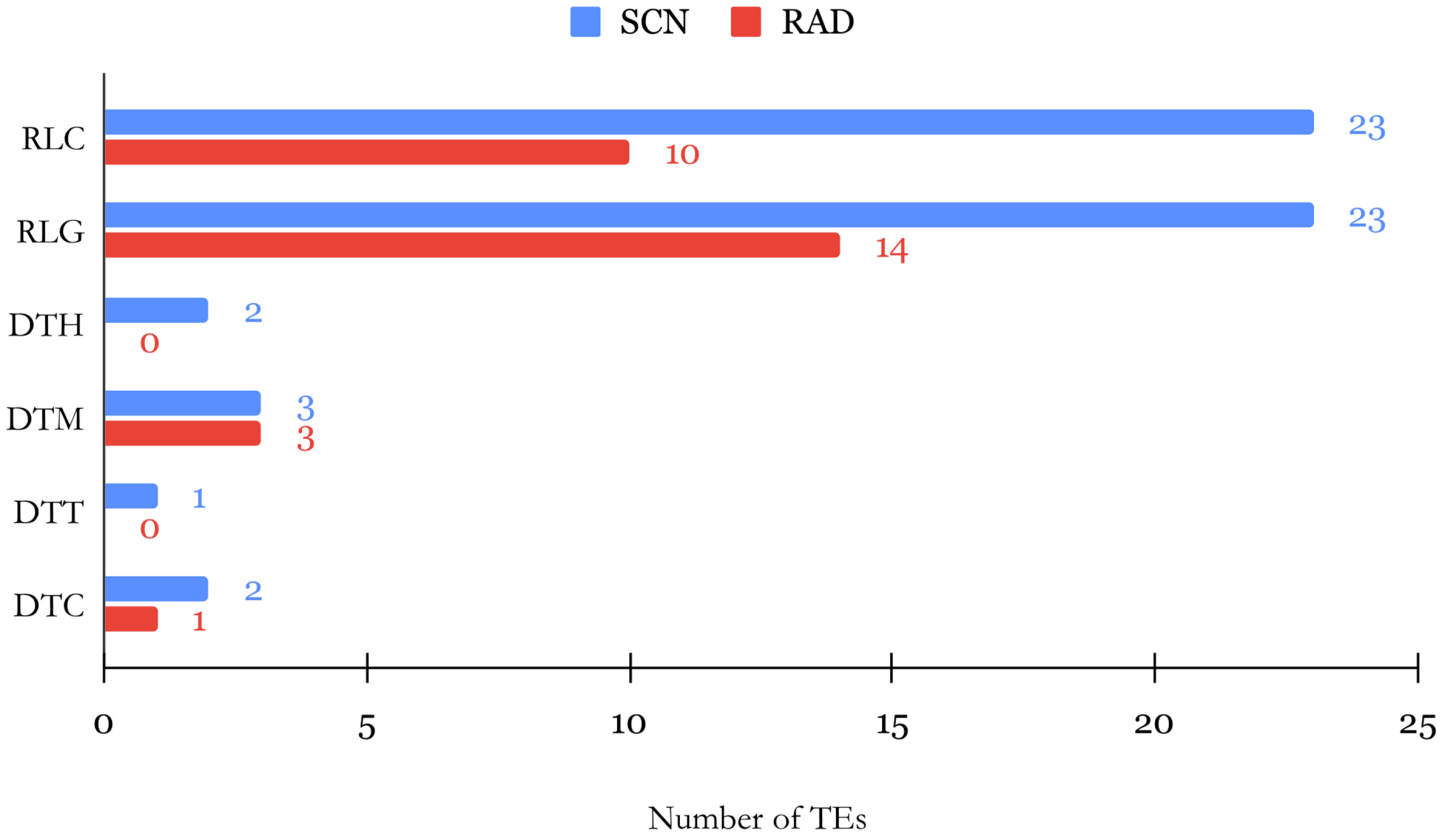
Barplot of the number of TEs by their family found within SCN- and RAD-associated lncRNAs.

The evolution and biological function of lncRNAs can be directly linked to the existence and composition of TE insertions within different sites of lncRNA sequences. TEs are repetitive mobile elements in the genome, and their composition in lncRNAs is proposed to affect the lncRNA functionality (Johnson and Guigó, 2014). Identification of TE composition of lncRNAs can thus unveil the link between structure and function of lncRNAs. Therefore, we used BLAST tools to compare the lncRNA transcripts with TEs in the SoyBase database. According to the classification system based on the transposition mechanism, sequence homology, and structural relationships suggested by (Wicker et al., 2007), several TE families belonging to class I (retrotransposons) and II (DNA transposons) were identified. Among lncRNAs of both studied data sets, LTR *Copia* (Ty1/copia; RLC) and LTR *Gypsy* (Ty3/Gypsy; RLG) families of TEs in class I were found in higher abundance compared to families grouped in class II (Figure 3). Furthermore, RLC with 23 TEs and RLG with 14 TEs were recognized as the largest families within SCN and RAD lncRNAs, respectively. In class II, *mutator* (DTM) and *CACTA* (DTC) families were identified with low abundance in both data sets, and no TEs belonging to *PIF/Harbinger* (DTH) and *Tc1–Mariner* families were detected among RAD lncRNAs.

### Functional Enrichment Analysis of lncRNA Potential Target Genes

**Figure 4:**
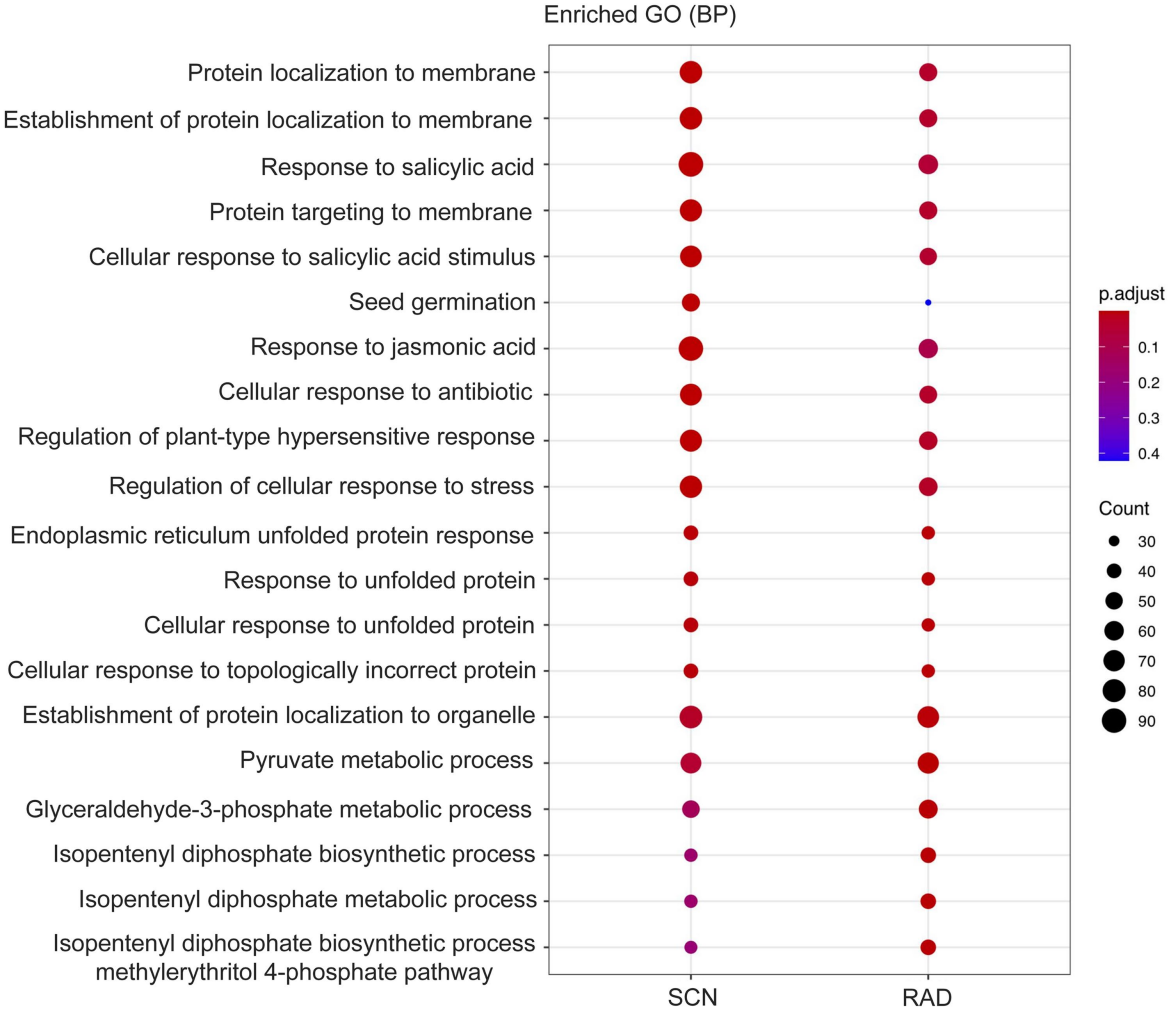
GO enrichment (BP: biological process) of genes located 100 Kbp upstream and downstream of lncRNAs.

**Figure 5:**
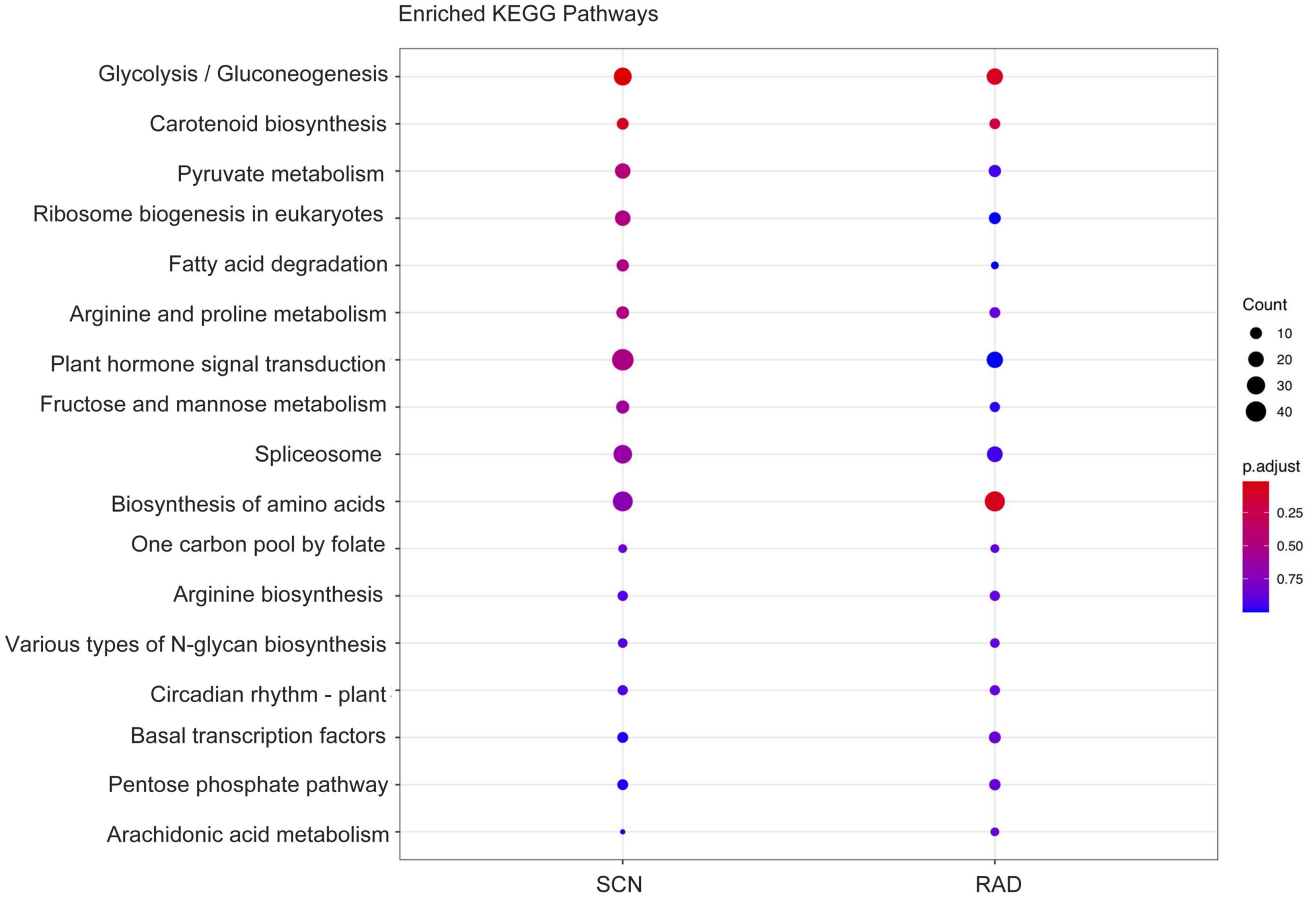
KEGG enrichment of genes located 100 Kbp upstream and downstream of lncRNAs.

To identify the function of the lncRNA cis-regulated potential target genes, GO enrichment and KEGG pathway enrichment analyses were conducted on the genes located 100 Kbp upstream and downstream of lncRNAs. GO enrichment analysis of cis-target genes revealed that the major PPN response-associated GO terms including, “response to salicylic acid,” “cellular response to salicylic acid,” “response to jasmonic acid,” “response to unfolded protein,” “regulation of plant-type hypersensitive response,” and “regulation of cellular response to stress” were found in both SCN data set and RAD. Among these key enriched BPs, “response to salicylic acid,” “response to jasmonic acid,” and “regulation of cellular response to stress” exhibited the highest number of lncRNA cis-regulated potential target genes in SCN data set. In general, the GO analysis demonstrated that lncRNA potential target genes in SCN data set and RAD were enriched similarly, but the number of genes enriched in a certain pathway was disparate. In RAD, “glyceraldehyde-3-phosphate metabolic process,” “isopentenyl diphosphate biosynthetic/metabolic processes,” and “isopentenyl diphosphate biosynthetic process, methylerythritol 4-phosphate pathway,” while in SCN data set, “seed germination” and “response to jasmonic acid” were the specifically enriched GO terms (Figure 4). According to the KEGG pathway enrichment analysis, “glycolysis/gluconeogenesis” and “carotenoid biosynthesis” were significantly enriched among upstream and downstream mRNAs under both SCN and reniform nematode infestation (Figure 5).

### Mining of DE-lncRNAs and ME-lncRNAs

To identify DE-lncRNAs, the differential expression analysis was carried out using DESeq2 package on the IDEAMEX web server, resulting in 42 and 111 potential DE-lncRNAs in SCN data set and RAD, respectively (Supplementary Tables S4 and S5). Among all identified lncRNAs, 141 lncRNAs were commonly shared between data sets (co-lncRNAs), in which 14 and 54 co-lncRNAs were DE-lncRNAs in SCN data set and RAD, respectively. The higher number of DE-co-lncRNAs in RAD can represent a more prominent role of lncRNAs in the reniform nematode defense response compared to SCN. To evaluate the expression pattern of DE-lncRNAs across all samples, a correlation analysis was performed using FPKM values. According to the heatmap of SCN DE-lncRNAs (Supplementary Figure S3), biological replicates of each time point were clustered together, suggesting the distinct expression profile of DE-lncRNAs under different time points. Comparing the expression level of DE-lncRNAs between infected and control samples displayed that the expression pattern of the DE-lncRNAs between N15 and control samples was highly distinct. The heatmap of RAD DE-lncRNAs indicated two completely segregated clusters, in which related replicates and samples were clustered together (Supplementary Figure S4).

**Figure 6:**
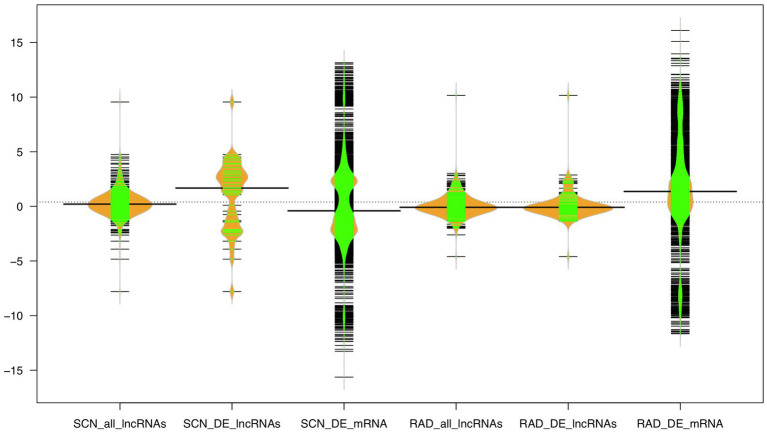
The expression distribution of the lncRNA and mRNA transcripts under SCN and reniform infestation. The width of the bean diagram represents the density (frequency) of the transcripts under a certain logFC (expression) value.

Furthermore, the expression distribution of all identified lncRNAs, DE-lncRNAs, and mRNAs was investigated based on log_2_ fold change (logFC) values across all samples in both data sets, where mRNAs showed a higher range of logFC (−15<logFC<15) compared to lncRNAs. In the SCN data set, the highest density of mRNAs was observed under the approximate logFC range of −1 to −3 and 2 to 3. A large proportion of lncRNAs displayed logFC between −2 and 2. Unlike all lncRNAs, DE-lncRNAs showed a higher expression distribution, and the highest density of DE-lncRNAs represented the approximate range of logFC between 2 and 4. In RAD, a large proportion of mRNAs appeared with −1<logFC<3, while the logFC of all lncRNA and DE-lncRNAs was similarly distributed in the range of −2 to 2 (Figure 6).

Using the MFSelector method, the ME-lncRNAs with strong monotonically changed patterns over time were separately identified in the SCN data set and RAD. The obtained ME-lncRNAs with the descending and ascending expression pattern are provided separately for SCN data set and RAD in Supplementary Tables S6 and S7. According to results, among 384 SCN-associated lncRNAs, 81 lncRNAs were expressed in descending order, among which three were DE-lncRNAs. In addition, 117 lncRNAs were expressed in ascending order, six of which were DE-lncRNAs. Among lncRNAs identified in the RAD database (283), 31 and 35 lncRNAs were expressed in ascending and descending order, respectively, within control samples. Four and seven ME-lncRNAs were also shared with DE-lncRNAs in the list of ascending and descending lncRNAs. Among infected RAD samples, we found 45 ME-lncRNAs expressed in descending order, of which six were also DE-lncRNAs. Furthermore, out of 36 lncRNAs expressed in ascending order, two ME-lncRNAs were found to be DE-lncRNAs.

### LncRNA Expression Study by Real-Time PCR

**Figure 7:**
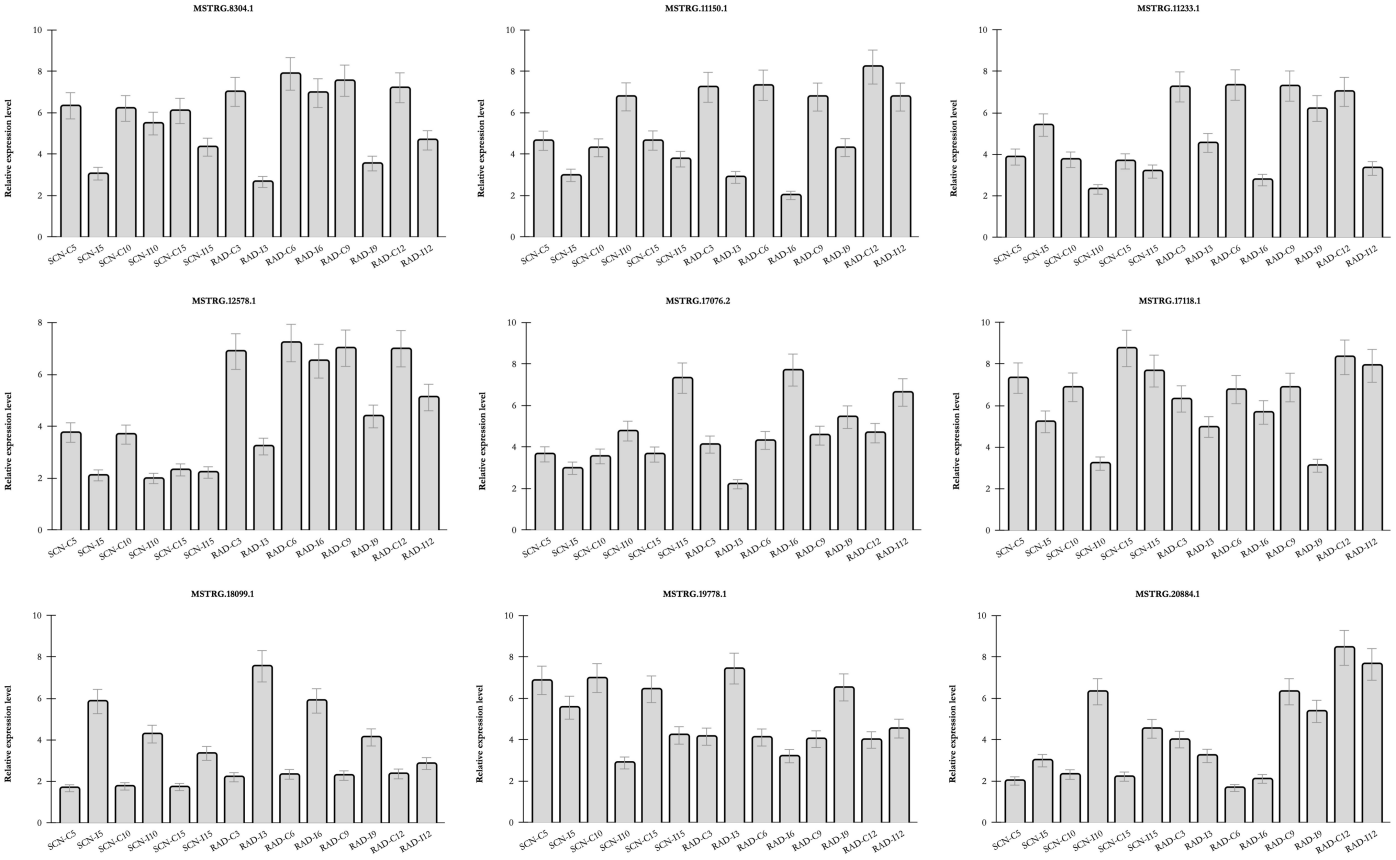
Relative expression of lncRNAs by qPCR Transcript levels of nine selected lncRNAs in roots of soybean plants under similar conditions to SCN data set and RAD and in three biological replicates. Bars show mean values of these replicates (+/− standard deviation).

The real-time PCR analysis was utilized to confirm the expression patterns of nine lncRNAs (Figure 7) including seven DE-lncRNAs from RAD and SCN data set, and two lncRNAs predicted to be target mimics of nematode-responsive miRNAs. LncRNAs were selected according to their target genes, whose pivotal roles were previously demonstrated in response to nematode infection. The results of examined lncRNAs by qPCR were almost concordant with the RNA-seq results, reflecting the accuracy of the RNA-seq data. Interestingly, some lncRNAs, including, MSTRG.18099.1, MSTRG.17118.1, MSTRG.8304.1, and MSTRG.12578.1, rendered a similar trend of expression in response to both SCN and reniform infection, suggesting a general lncRNA-mediated defense response in soybean against PPNs. On the other hand, some lncRNAs exhibited nematode type-dependent regulatory behavior. For instance, in response to SCN, MSTRG.20884.1 was upregulated over different time points, while under the reniform treatments, it was downregulated. Contrariwise, MSTRG.19778.1 appeared with a negative regulatory role under SCN infection, whereas, in response to reniform nematode, its expression indicated a positive regulatory role. In addition, the expression pattern of two lncRNAs predicted to be target mimics of nematode-responsive miRNAs was validated by qPCR. These lncRNA-miRNA pairs, MSTRG.17076.2-gma-miR156aa/z and MSTRG.11150.1-gma-miR319p, should be further investigated for their potential role in the regulation of response to nematodes in soybean.

### Predicting the lncRNAs Interaction With DEGs

In addition to lncRNA-mediated transcriptional regulation of the adjacent genes (cis-regulated target genes), lncRNA-mediated regulation is also carried out by base pairing with complementary mRNAs (Yan et al., 2020). Hence, we employed LncTar^3^ to predict lncRNA trans-regulated target genes. According to the results, a total of 23 and 32 DEGs were found to be potential lncRNA target genes in SCN data set and RAD, respectively. Regarding the annotation of these identified DEGs (DE target genes), the potential PPN-responsive genes, including, Glyma.01G007100.1 (*ABS3*), Glyma.01G008200.1 (*ABCB15)*, Glyma.10G269400.5 (*BBX27*), Glyma.10G269700.1 (*BAT1*), Glyma.13G029200.1 (*scpl40*), Glyma.13G210100.1 (*AT1G49330.1*), and Glyma.19G106700.1 (*XTH5*), were identified in SCN data set. Similarly, the DE target genes were found in RAD to be involved in the soybean immune responses to PPNs. These DEGs included Glyma.02G059400.1 (*GATL2*), Glyma.02G248500.1 (*ATEXP4*), Glyma.07G070200.1 (*CDKB2;2*), Glyma.07G089000.2 (*VIL1*), Glyma.08G091500.1 (*GAD*), Glyma.13G069900.1 (*GASA14*), and Glyma.14G075800.2 (*EXA1*). Among the DE target genes detected in SCN data set, no annotation was found for Glyma.10G052400. Additionally, in RAD, Glyma.02G265200, Glyma.02G265300, and Glyma.02G265500 were not annotated. These genes can potentially be specific players of the PPN immune response in soybean.

The expression profile of SCN DE targets across all samples displayed that infected samples were clustered together, while control samples were separated (Supplementary Figure S5). Similar to lncRNAs, the heatmap of RAD-DE targets displayed two distinct clusters in which related samples were clustered together (Supplementary Figure S6). A comparison between the expression pattern of the potential PPN-associated DE targets and their regulatory lncRNAs exhibited an opposite pattern in infected samples compared to the controls. For instance, this negative correlation was observed between Glyma.10G052500.1 (*CRPK1*) and MSTRG.18338.1 in N5 compared to control, and between Glyma.10G269400.5 (AT1G68190.1; B-box zinc finger family protein) and MSTRG36158.1, Glyma.10G269700.1 (*BAT1*) and MSTRG.4058.1, and also between Glyma.13G029200.1 (*scpl40*) and MSTRG.27380.1 in N10, compared to control samples. Besides, the opposite expression pattern between Glyma.11G091400.1 (*ATFP6*) and MSTRG.36158.1 and MSTRG.36243.1 lncRNAs, Glyma.13G210100.1 (AT1G49330.1; hydroxyproline-rich glycoprotein family protein: *HRGP*) and MSTRG.11812.1, MSTRG.11233.1, and MSTRG.36158.1, and between Glyma.19G106700.1 (*EXGT-A4*) and MSTRG.36158.1, demonstrated the strong correlation between the aforesaid lncRNAs and their related targets. The functional relationship could be inferred from this association, although further analysis is needed to validate. Likewise, in the RAD, these opposite expression patterns were deciphered between lncRNAs and their PPNs-related DE targets. For instance, the probable lncRNA-mediated regulation was detected for Glyma.02G248500.1 (*ATEXP4*; by MSTRG.12578.1), as well as for Glyma.08G091500.1 (*GAD*; regulated by MSTRG.38154.1), and Glyma.13G069900.1 (homolog of AT5G14920.1; Gibberellin-regulated family protein; regulated by MSTRG.19778.1) in the infected samples compared to the control.

### Prediction of miRNA Mimics Using lncRNAs

To decipher the crosstalk between lncRNAs and miRNAs in soybean against nematode infection, lncRNAs that may act as eTMs were predicted using psMimic algorithm (Wu et al., 2013; Supplementary Table S8). According to the results, a total of 15 lncRNAs identified in the SCN data set were predicted to be potential eTMs for 17 miRNAs. Among them, only one lncRNA (MSTRG.26464.1) was found to be DE-lncRNA. Searching for eTMs in RAD revealed six lncRNAs as potential eTMs for seven miRNAs. Four lncRNAs were among DE-lncRNAs (MSTRG.2711.1, MSTRG.17076.2, MSTRG.26588.1, and MSTRG.26609.1). Our results showed the presence of eTM sequences within some important classes of conserved miRNAs including miR156, miR319, and miR396, whose crucial role has been illustrated in response to nematode invasion (Hewezi et al., 2012; Zhao et al., 2015; Yin et al., 2019). In this study, we identified several eTM hits with different family members of miR156 (gma-miR156aa, gma-miR156z, gma-miR156g, and gma-miR156b in SCN data set; gma-miR156aa, and gma-miR156z in RAD), miR319 (gma-miR319p in SCN data set and RAD), and miR396 (gma-miR396a-3p in SCN data set), which can manifest the function of corresponding lncRNAs regarding PPN infection.

In further analysis, using psRobot toolbox, we identified 177 and 464 putative unique target genes for seven and 16 miRNAs in SCN data set and RAD, respectively, that previously exhibited mimicry with lncRNAs (Supplementary Table S9). Among predicted target genes identified in SCN data set, genes, such as Glyma.01G050100 (*AT-EXP1*; gma-miR1533 target gene), Glyma.06G259800 (*DSC1*; gma-miR1533 target gene), Glyma.14G089090 (*AtGRF1*; gma-miR1535a target gene), Glyma.17G192000 (*AAP6*; gma-miR156g target gene), and Glyma.09G270000 (*SCR*; gma-miR9722 target gene), were found as the putative nematode-related genes. In RAD, the major target genes associated with nematode stress response included Glyma.02G255800 (*AUX1*; gma-miR5369 target gene), Glyma.20G047600 (*ATMYB33*; gma-miR319p target gene), and Glyma.14G078600 (*AOS*; gma-miR9725 target gene).

### LncRNA-mRNA Co-expression Study Under PPN Infestation

In the current study, WGCNA analysis was applied to study the co-expression of lncRNAs and mRNAs in response to two main destructive PPN species, *H. glycines* and *R. reniformis*, in soybean. Using hierarchical clustering of 1-TOM and the DynamicTree Cut algorithm on 4,109 unique DEGs and 51 DE-lncRNAs identified in SCN data set, 21 modules with distinct colors were identified. The dynamic clustering modules were merged based on the module eigengene (the most representative gene expression in a module) similarity (≥0.8) and reduced to eight modules ranging in size from 128 to 976 genes in each module (Table 2). A similar analysis was performed on 2,400 unique DEGs and 111 DE-lncRNAs of the RAD, resulting in 14 modules that were labeled with different colors. Modules with highly correlated eigengenes were merged and lessened to 12 modules with a size range of 61 to 594 genes in each module (Table 2). Among 12 identified co-expression modules, the brown ones with 50 lncRNAs encompassed the highest number of DE-lncRNAs. After that, the green, black, and magenta modules with 15, 10, and 8 lncRNAs were, respectively, recognized as modules with the maximum number of DE-lncRNAs.

**Table 2:** The number of DEGs and DE-lncRNAs in each co-expression module in SCN data set and RAD.

	Total num.	DEG num.	DE-lncRNA num.
SCN data set
Cyan	976	968	8
Green	892	880	12
Grey	128	127	1
Light cyan	432	424	8
Midnight blue	137	135	2
Purple	160	159	1
Red	779	765	14
Royal blue	656	651	5
RAD
Black	93	83	10
Blue	395	392	3
Brown	285	235	50
Green	118	103	15
Green yellow	66	64	2
Grey	594	589	5
Magenta	554	546	8
Pink	85	83	2
Purple	80	77	3
Red	111	107	4
Salmon	61	56	5
Tan	69	65	4

The dynamic modules and the merged dynamic modules have been displayed in Supplementary Figure S7 and S8 for SCN data set and RAD, respectively. Based on this analysis, 128 transcripts (including 127 DEGs and one DE-lncRNAs) obtained from SCN data set and 594 (including 589 DEGs and five DE-lncRNAs) from RAD were not assigned to any co-expressed module and classified into the grey module. The red module in SCN data set with 14 DE-lncRNAs, and the brown module in RAD with 50 DE-lncRNAs encompassed the largest number of DE-lncRNAs.

Further investigation among genes and lncRNAs of each module detected novel relationships between DE-lncRNAs and nematode-associated DEGs. For instance, in the red module of the SCN data set, the potential lncRNA/mRNA crosstalk-mediated modification of cell wall structure in response to SCN infection was identified through co-expression of 14 identified DE-lncRNAs with already known wall-modifying DEGs including a *HRGP* and xyloglucan endotransglycosylase/hydrolase43 (*XTH43*). Furthermore, in the current study, the potential lncRNA/mRNA crosstalk-mediated signaling cascade against nematode infection was detected between lncRNAs and leucine-rich repeat receptor-like protein kinases (*LRR-RLKs*), CBL-interacting protein kinase (*CIPK*), and BRI1-associated receptor kinase (*BAK1*) as neighboring signal transduction-related DEGs under nematode invasion. In green module, several associations were detected between 12 DE-lncRNAs and genes including a RING/U-box superfamily gene, *HRGP*, an aldolase-type TIM barrel family gene, laccase7 (LAC7), a copper transport protein family gene, auxin response factor8 (*ARF8*), a MYB domain-containing gene, and several heat shock protein (*HSP*) genes. The co-expression of eight DE-lncRNAs with nitrite reductase and ACC oxidase genes in the lightcyan module, two DE-lncRNAs with galactinol synthase, a drought-induced gene, NAC and MADS-box family genes in the midnight blue module, eight DE-lncRNAs with the pectin lyase-like superfamily gene and UDP-Glycosyl transferase in the cyan module, five DE-lncRNAs with UDP-D-galactose 4-epimerase5, plasma membrane intrinsic protein (*PIP*), WUSCHEL-related homeobox (*WOX*), and ABC transporter family gene in the royal blue module, and finally one DE-lncRNAs with several *EXP*s in the purple module unraveled the possible association between lncRNAs and important pathogenesis-related genes in soybean. Similar to SCN data set, In RAD, the novel associations among DE-lncRNAs and the major nematode-responsive DEGs were elegantly recognized in all 12 identified modules. For instance, the potential associations were observed between 50 lncRNAs and nuclear transport factor 2 family gene (*NTF2*) and RING/FYVE/PHD zinc finger superfamily protein in the brown module. Besides, in the green module, the potential relationship was detected between 15 DE-lncRNAs and neighboring nematode resistance-related genes such as *GRAS*, *bZIP*, *ABC1*, and *MEKK* family members, as well as glutathione synthetase2 (*GSH*). In the rest of the modules, DEGs including glycosyl hydrolases family gene (in the black module), Aquaporin-like superfamily gene (in the blue module), signal peptide peptidase-like2 gene (in the greenyellow module), and CAP (cysteine-rich secretory proteins, antigen5, and pathogenesis-related1 protein) superfamily gene (in the magenta module) were some of the nematode response-associated genes identified in RAD.

**Figure 8:**
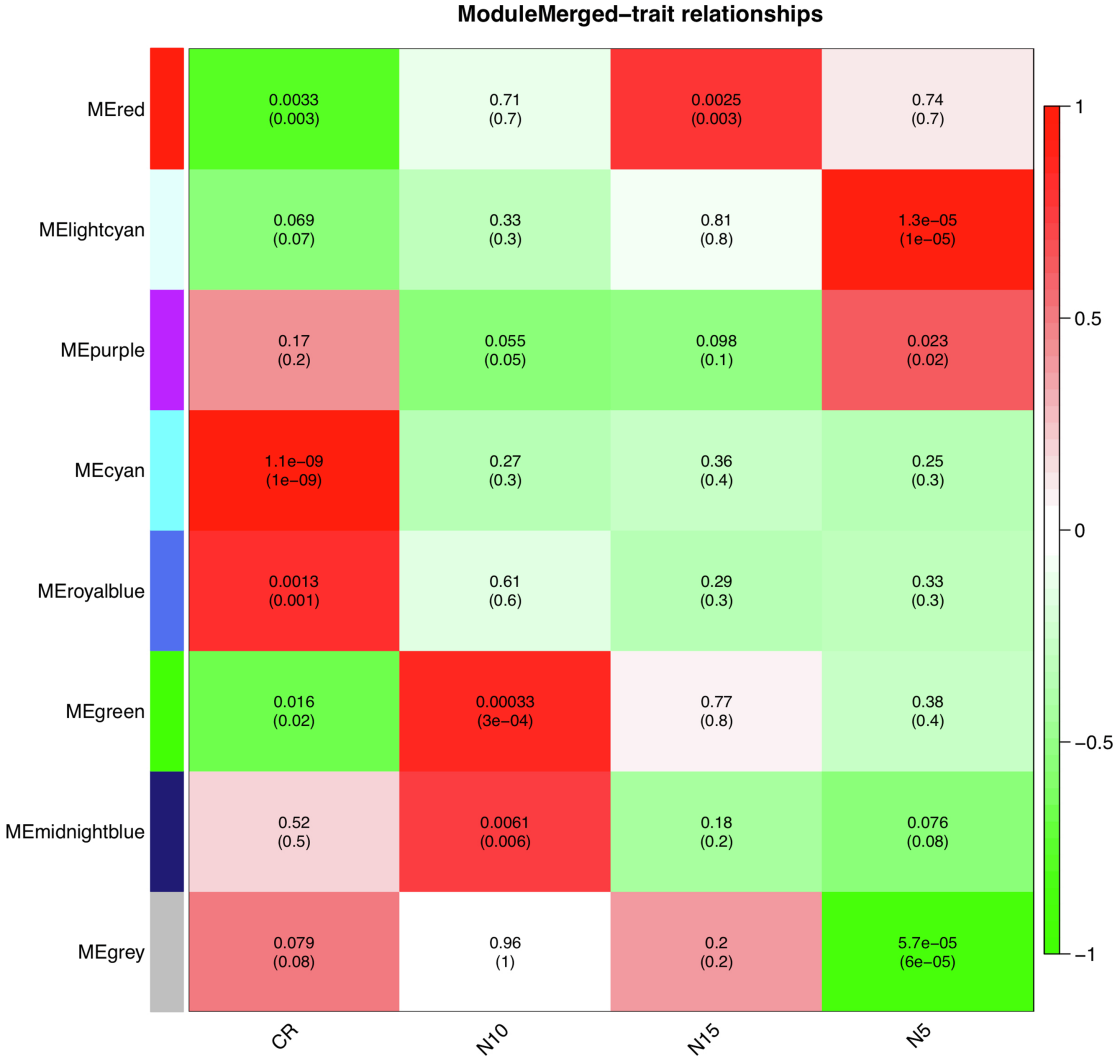
The heatmap of module-traits relationships in SCN data set. The heatmap illustrates correlations between module eigengenes and dpi traits. The numbers represent Pearson’s correlation coefficients and *p*-values. The color legend represents the strength and direction of correlations.

**Figure 9:**
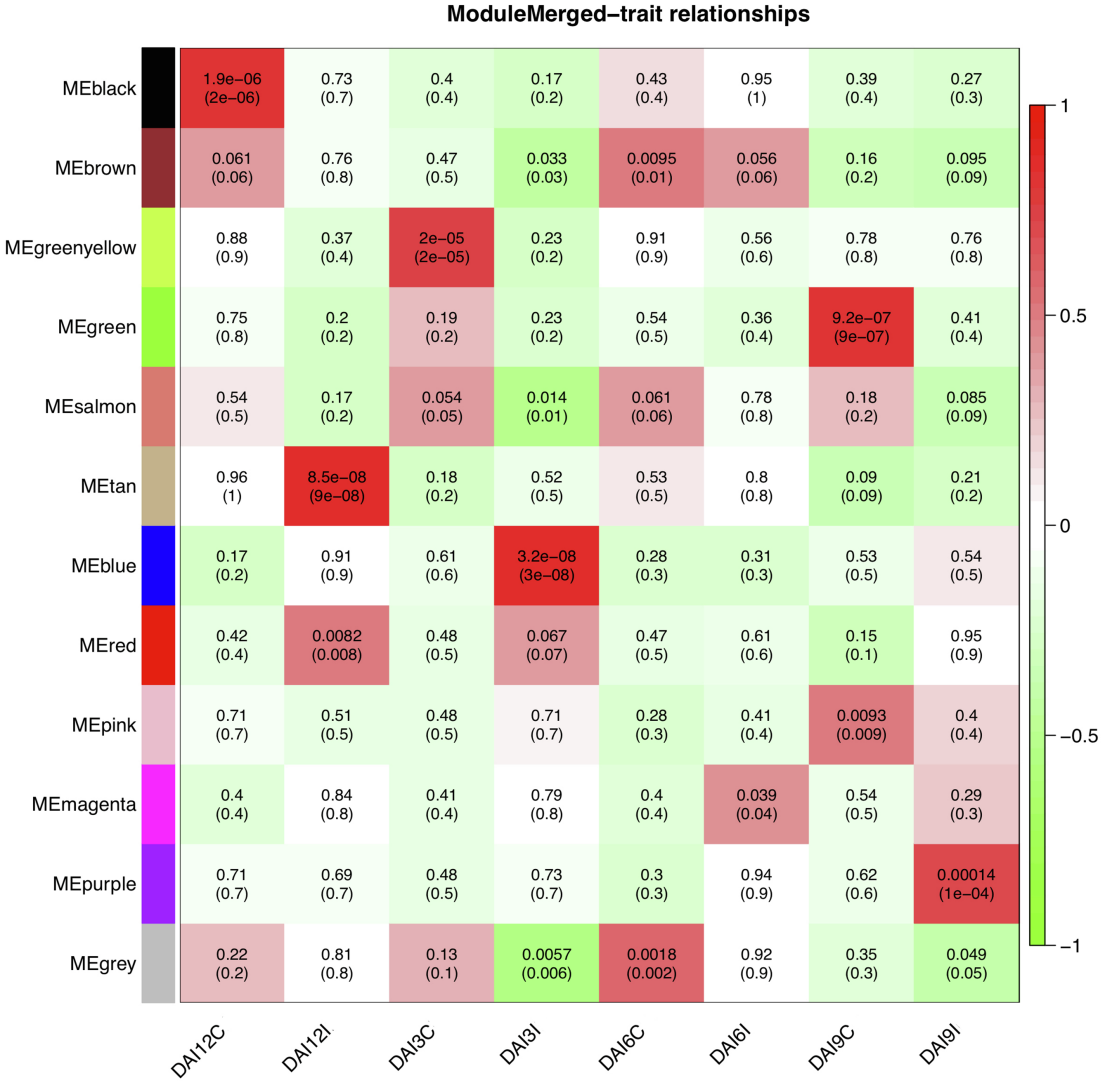
The heatmap of module-traits relationships in RAD. The heatmap illustrates correlations between module eigengenes and DAI traits. The numbers represent Pearson’s correlation coefficients and *p*-values. The color legend represents the strength and direction of correlations.

To find significantly correlated modules with the nematode infestation response, Pearson’s correlation coefficients between module eigengene values in each module and the dpi traits (as the individual gene expression values under CR, N5, N10, and N15 treatments related to SCN data set) were calculated. According to the heatmap of the module-trait relationship (Figure 8), significantly correlated modules generally displayed low correlation coefficient values. The green module was correlated negatively with CR and positively with N10. The red module was also inversely correlated with CR while positively correlated with N15. This positive correlation suggests the presence of upregulated eigengenes under N10 and N15 treatments in the green and red modules, respectively. The purple module had a significant positive correlation with N5 while conversely had a significant negative correlation with N10. These opposite correlation coefficient values can represent the remarkable changes in the gene expression profiles between N5 and N10 treatments. Besides, a similar analysis was performed between module eigengenes and DAI traits (DAI12C, DAI12I, DAI3C, DAI3I, DAI6C, DAI6I, DAI9C, and DAI9I) in RAD (Figure 9). In this data set, the salmon module positively correlated with DAI3C while negatively correlated with DAI3I. This negative correlation represents the presence of downregulated eigengenes under DAI3I treatment. The brown module was inversely correlated with DAI3I, as well. The significant positive correlation observed in the modules including tan (under DAI12I), blue (under DAI3I), red (under DAI12I), magenta (under DAI6I), and purple (under DAI9I) demonstrated the presence of upregulated genes that can regulate soybean immune response to PPNs.

## Discussion

**Figure 10:**
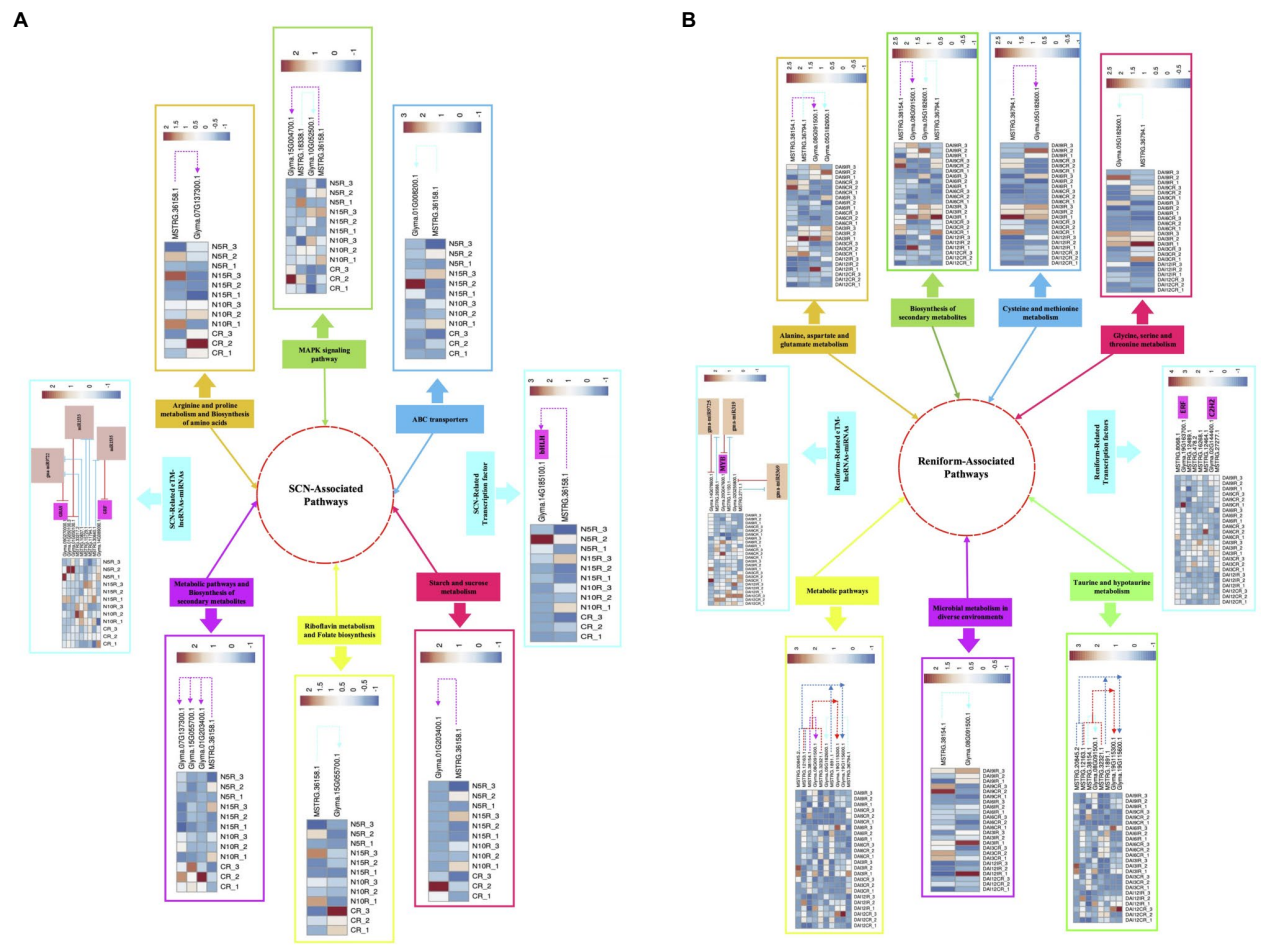
The schematic representation of probable regulatory mechanism in response to PPNs infection in soybean. The proposed model illustrating the SCN-associated pathways **(A)** and reniform-associated pathways **(B)** in which lncRNAs regulate their nematode-responsive target gene expression (including protein-coding genes and TFs). Additionally, in this model, the involvement of lncRNAs in miRNA-mediated regulation of gene expression under nematode infection has been displayed. The heatmaps represent the expression patterns of lncRNAs and their potential target genes in each sample.

PPNs are the most ruinous pathogens in soybean that lead to serious damages and severe yield loss (Bandara et al., 2020). Due to their sophisticated parasitizing strategies, comprehensive assessments are required to get meticulous insight into the underlying resistance mechanisms in soybean. In recent years, novel regulatory pathways involved in plant-nematode interplay have been revealed through genome-wide characterization of nematode-responsive lncRNAs (Li et al., 2018c). In addition, novel regulatory layers of gene expression in several plant species have been identified *via* unraveling the interaction between miRNAs and lncRNAs (Meng et al., 2021). Although some of the potential pathways, networks, and associated components that are involved in nematode response and modulate nematode resistance in soybean have been detected through various transcriptome analyses, lncRNA-mediated nematode defense responses are poorly understood in this species. Hence, in the present study, we undertook genome-wide identification of lncRNAs linked with two nematode species (SCN and reniform nematode) followed by (1) assessment of the evolutionary conservation of identified lncRNAs, (2) characterizing TE content of lncRNAs, (3) the differential expression analysis, (4) characterization of the ME-lncRNAs with a strong monotonic pattern among different time points, (5) functional annotation of lncRNAs *via* prediction of potential cis- and trans-target genes, (6) detection of the crosstalk between lncRNAs and miRNAs under nematode invasion, and finally, (7) construction of soybean’s lncRNA-mRNA co-expression networks using WGCNA analysis. At the end, the probable regulatory mechanisms of lncRNAs and their potential and well-studied target genes in response to SCN and reniform infection in soybean were proposed in network models (Figure 10).

Upon application of a multi-step pipeline, lncRNAs and DE-lncRNAs were detected in SCN data set and RAD, respectively. The number of lncRNAs, co-lncRNAs, DE-lncRNAs, and co-DE-lncRNAs could reveal the specific function of lncRNAs in response to particular nematode species, additionally highlight SCN-/RAD-associated lncRNAs for further confirmation-based experiments. The conservation of lncRNAs was further evaluated using homology search against the three plant lncRNA sequences database; CANTATAdb, GreeNC, and PLncDB, and recently reported lncRNAs in soybean (Golicz et al., 2018; Lin et al., 2020). The results of this analysis deciphered the potential role of MSTRG.12060.1 in SCN immune response. Regarding the functional annotation of lncRNAs provided by (Golicz et al., 2018), the involvement of MSTRG.12060.1 in the benzene-containing compound metabolic process was predicted. This BP is recognized as the defense-related pathway in wheat under cereal cyst nematode (CCN; *Heterodera avenae*) infestation (Kong et al., 2015). Moreover, the potential role of MSTRG.17157.1 in RAD immune response was detected in the current study. This lncRNA was previously suggested to have a role in the endocytosis process (Golicz et al., 2018). Once the pathogenic organisms invade host cells, the endocytosis process is activated to induce signal transduction through surface-localized immune receptors. Thereupon, the polarized secretion delivers antimicrobial compounds, components of the cell wall, and defense proteins to invasion sites (Gu et al., 2017). Therefore, MSTRG.17157.1 might participate in plant immune activation in the early hours after nematode invasion.

In this study, the identified lncRNAs in SCN have shown to contain 14.1% of TEs sequences with LTR *Copia* and LTR *Gypsy* as the most abundant families (42.6%). In RAD, 9.9% of lncRNAs contained TE sequences highlighting an overrepresentation of LTR *Gypsy* (50%) followed by LTR *Copia* (35.7%). TE-derived sequences proportion of lncRNAs detected in this study was compared with other species, and their contribution found to be very variable depending on the considered lncRNA under different conditions. Some plant species represented the remarkable proportion of TE-lncRNAs. For instance, 65% of *Zea mays* lncRNAs (Lv et al., 2019) and 53% of *Oryza sativa* lncRNAs (Zhou et al., 2021) contained sequences that appear to be derived from TEs. On the other hand, some plant species, such as *Brachypodium distachyon* with 8% (De Quattro et al., 2017), *Aegilops tauschii* with 19%, and *Triticum urartu* with 27% (Pieri et al., 2018), exhibited less proportion of lncRNAs which can be attributed to TEs. Regarding these findings, these differences can demonstrate that TEs are likely to be leading actors of the swift evolutionary turnover of lncRNAs among various species (Etchegaray et al., 2021). Despite the differences between the species, LTR *Gypsy* elements contribute TE-lncRNAs at a highest proportion. For example, LTR *Gypsy* with 62.5% in *Zea mays*, 59% in *Oryza sativa*, and 80.5% in *Brachypodium distachyon* reflected its predominance compared to other TE families. Unlike protein-coding genes, in which a newly discovered loci can be easily annotated through homologous genes of known function, in lncRNAs, due to lack of characterized homologs, the annotation is delimited (Smith and Mattick, 2017; Golicz et al., 2018). Hence, we implemented the functional enrichment analysis of lncRNA cis-target genes to explore the potential functions and relevant regulatory mechanisms of lncRNAs. GO enrichment analysis of neighboring genes detected enrichment of BPs that are involved in nematode infection responses, among which, “response to salicylic acid” and “response to jasmonic acid” BPs were recognized as significantly enriched nematode response-related pathways. Nematodes are able to trigger complicated alterations in hormone biosynthetic and signaling pathways (Kammerhofer et al., 2015). On the other hand, crosstalk between different hormone signaling pathways leads to immune responses in plants (Klessig et al., 2018), and both salicylic acid (SA)- and jasmonic acid (JA)-dependent systemic acquired resistance (SAR) are shown to be induced in nematode-infected *Arabidopsis thaliana* (Arabidopsis) roots (Hamamouch et al., 2011). Several studies indicated that JA and SA pathways shape the backbone of the plants’ immune signaling network (Mbaluto et al., 2021). It is indicated that the induction of resistance to phytonematodes in tomato is correlated with the SA content (Zinov’eva et al., 2011). Additionally, the probable involvement of the endogenous SA has been remarked in Mi-1-mediated defense responses and in lesion formation in sites directly exposed to root-knot nematode (RKN) invasion (Molinari and Loffredo, 2006). Likewise, the functional annotation of DEGs in sweet potato (Lee et al., 2021) and in cotton (Kumar et al., 2019) demonstrated “response to salicylic acid” as the key enriched BP against nematode infestation. The role of JA, as another plant signaling compound, has been investigated upon nematode infection in different plant species (Hamamouch et al., 2011; Fan et al., 2015; Regis, 2015; Singh et al., 2020). JA-mediated tolerance against RKN infection is reported through alteration in the photosynthetic and antioxidative defense mechanisms in tomato plants (Bali et al., 2018).

In the current study, “endoplasmic reticulum unfolded protein response” was also found as one of the enriched GO terms. Different stressors, including PPNs, usually lead to the accumulation of unfolded proteins in the endoplasmic reticulum of plant cells, following which the drastic need for protein folding is signaled (Kandoth et al., 2011) and the unfolded protein response (UPR) as a conserved stress response is activated (Bao and Howell, 2017). “Pyruvate metabolic process” was recognized as another significantly enriched nematode response-related pathways. Although the obvious involvement of pyruvate metabolic process has not reported in response to phytonematodes, it has been displayed that pyruvate directly and indirectly associated with diminishing oxidative damages induced by various stresses (Savchenko and Tikhonov, 2021). “Isopentenyl diphosphate metabolic/biosynthetic processes” were another detected enriched BPs against PPNs infestation. Isopentenyl diphosphate (IPP) has been identified as the precursor for terpenoids biosynthesis which is involved in plant direct defense (Vieira et al., 2019). The inhibitory role of these metabolites has been found against the RKN larvae (Ohri and Pannu, 2009). GO analysis was revealed enrichment of “establishment of protein localization to membrane.” This term contains direct descendants terms including, “protein targeting to membrane,” “protein transport out of plasma membrane raft,” and “protein transport into membrane raft” which contribute in defense response through major membrane transport proteins such as ATP-binding cassette (ABC) proteins, identified as the predicted target for MSTRG.36158.1 in this study. The members of this protein family found to be involved in detoxification pathways to reduce toxic compounds through pumping of PPN’s xenobiotic metabolites. These proteins have also been also recognized as potential target to control PPNs (Kooliyottil et al., 2020).

Results of the KEGG enrichment analysis indicated the significant enrichment of “glycolysis/gluconeogenesis” among lncRNA’s neighboring protein-coding genes. Since the female nematodes need a large amount of energy, genes involved in glycolysis/gluconeogenesis are upregulated during nematode feeding. This has been reported in soybean roots infected by SCN (Matthews et al., 2011). The “carotenoid biosynthesis” was another significantly enriched pathway under both SCN and reniform nematode invasion. A positive correlation between rice susceptibility to RKN and accumulation of chlorophyll/carotenoid precursors was detected (Kyndt et al., 2017). The authors hypothesized that accumulation of chlorophyll and carotenoid precursors might be advantageous to nematode infection, which leads to enhanced susceptibility of rice against RKN.

To elucidate the interaction between lncRNAs and DEGs, trans-regulated target genes were predicted using lncTar, and their expression profile was compared with lncRNA’s expression profile across all samples. Among predicted DE targets, there were genes whose expression pattern was opposite to their complementary lncRNAs. For instance, a protein kinase superfamily (Glyma.10G052500) gene and a B-box zinc finger family (Glyma.10G269400) gene were detected as DE targets, which might be negatively regulated by MSTRG.18338.1 and MSTRG.36158.1, respectively. Rambani et al. (2015) showed that SCN infection induces the formation of multinucleated feeding sites or syncytium, whose etiology includes DNA methylation and expression changes of genes. In detail, genes involved in signal transduction and regulation including B-box zinc finger and protein kinases were found as syncytium differentially methylated genes (Rambani et al., 2015). Regarding these observations and the results of SCN data set analysis, we suggest that both SCN-dependent differential methylation and lncRNA-mediated regulation can modulate the expression of protein kinase superfamily and B-box zinc finger family genes.

Bidirectional amino acid transporter1 (*GmBAT1*) was identified as another DE target that was potentially suppressed by MSTRG.4058.1. Due to the lack of aspartate kinase in nematodes, they are not capable of synthesizing lysine, threonine, methionine, and isoleucine amino acids. Hence, they have to supply amino acids from host plants by manipulating their metabolism (Frey, 2019). Arabidopsis *AtBAT1* has been detected to export glutamate and lysine (Guo et al., 2019). Until now, there is no evidence about how nematodes reprogram *BAT1* expression to deliver required amino acids. The downregulation of this gene in infected samples (N5 and N10), may imply that MSTRG.4058.1 (suppression of *BAT1*) can inhibit providing essential amino acids to SCN’s feeding sites. Serine carboxypeptidase (SCP)-like40 (*GmSCPl40*)/MSTRG.27380.1 is another potential trans-target/lncRNA pair. The SCP family encodes a disparate range of enzymes involved in the degradation and processing of proteins, and several SCP proteins are involved in the production of secondary metabolites (Fraser et al., 2005). The role of SCPs in the parasitic process of the *Radopholus similis* PPN was functionally investigated by (Huang et al., 2017), and the notable induction of SCP was reported by (Puthoff et al., 2007) across different time points in SCN-inoculated soybean roots (Puthoff et al., 2007; Huang et al., 2017). In the current study, the significant upregulation of *SCPl40*, probably mediated by MSTRG.27380, in N10 compared to control samples, suggests the probable positive role of *SCPl40* in soybean immune response to SCN. *HRGP* (target of MSTRG.11233.1), as the essential cell wall-associated protein, was an overexpressed gene in N15 compared to N5. The involvement of *HRGP* in cell wall resistance and inhibition of pathogen’s spatiotemporal ramification through arresting pathogen at the invasion site has been reported in multiple studies (Deepak et al., 2010; Xie et al., 2011; Hijazi et al., 2014; Rashid, 2016). Additionally, the role of *HRGP* in conferring SCN resistance to soybean plants has been displayed (Zhao et al., 2017). In our study, the *HRGP*/MSTRG.11233.1 interaction can approve lncRNA-mediated cell wall resistance against SCN in soybean. Xyloglucan endotransglucosylase/hydrolase (*XTH*) is recognized as a cell wall-modifying gene, and its downregulation was detected in SCN-colonized root pieces (Tucker et al., 2010). In line with this, the descending expression pattern was found for this gene over the course of time series. Interestingly, elevated expression was observed for MSTRG.36158.1 (potential regulatory lncRNA of *XTH*) over time up to 10days after infection. *XTH5* has been turned out to be involved in the construction of cell wall during growth and differentiation through cleavage of xyloglucan polymers, a substantial constituent of the primary cell wall (Worthington et al., 2021). Since improving the root cell wall resistance is one of the defense mechanisms to restrict nematode’s stylet penetration into cells (Sato et al., 2019), we can assume that modification of cell wall structure might occur through MSTRG.36158.1-mediated suppression of *XTH5*.

Similar to the SCN data set, in RAD, the interplay between lncRNAs and DE target genes was investigated. Among target genes, homolog of Arabidopsis Expansin4 (*AtEXP4*) was found to be potentially regulated by MSTRG.12578.1. Expansin genes encode cell wall loosening agents and regulate root growth by influencing the non-covalent bonds between cellulose and hemicellulose (Samalova et al., 2020). Expansins not only participate in cell wall modification but also appear to induce and suppress host defenses (Ali et al., 2015). The induced expression of soybean homolog of *AtEXP4* by MSTRG.12578.1 in the infected samples compared to control may explain the effect of reniform nematode on lncRNA/*ATEXP4* expression reprogramming. Another PPN-associated DE target gene in RAD, which might be regulated by MSTRG.38154.1, was glutamate decarboxylase (*GAD*). This gene positively regulates the resistance against the northern RKN in tobacco (McLean et al., 2003). This gene is involved in the synthesis of γ-aminobutyric acid (GABA), whose metabolism is activated following the invasion of various groups of pathogens and pests. The inhibitory effect of GABA on the neuronal transmission of insects as well as its regulatory role on the hypersensitive response has proved its capacity in host defense response (Tarkowski et al., 2020). In our study, *GAD* appeared with an upward expression trend over the course of time series. This result suggests the positive regulatory role of *GAD* in response to the reniform nematode in soybean.

Apart from the independent regulatory role of each miRNA and lncRNA on mRNAs, lncRNAs can also act as eTMs to neutralize the gene silencing effect of miRNAs. The lncRNA-miRNA interaction can modulate the transcriptome under different conditions (López-Urrutia et al., 2019). In the current study, among target genes predicted by psRobot several genes were found to be validated by degradome sequencing in different studies (Shamimuzzaman and Vodkin, 2012; Xu et al., 2016; Zhou et al., 2020) including, Glyma.12G032600 (gma-miR319 target gene), Glyma.02G121300 (gma-miR156 target gene), Glyma.02G177500 (gma-miR156 target gene), Glyma.05G019000, (gma-miR156 target gene), Glyma.06G168600, Glyma.17G080700 (gma-miR156 target gene), Glyma.07G256700 (gma-miR1535 target gene), and Glyma.13G159700 (gma-miR396a-3p target gene). In addition to deciphering crosstalk between conserved nematode-related miRNAs (miR156, miR319, and miR396) and lncRNAs, the novel potential regulatory components, including MSTRG.11794.1/MSTRG.15729.1-gma-miR1533, MSTRG.30640.1-gma-miR1535a, and MSTRG.10837.1/MSTRG.33217.2-gma-miR9722 under SCN infection, were introduced. According to the psRobot results, gma-miR1533 is predicted to regulate soybean *EXP1*. This gene, as one of the cell wall-modifying genes under nematode infection, participates in nematode-induced syncytia formation in *Nicotiana benthamiana* roots (Liu et al., 2016). Homolog of Arabidopsis growth-regulating factor1 (*AtGRF1*) is predicted to be targeted by gma-miR1535a. The pivotal role of *AtGRF1* has been demonstrated in the reprogramming of root cells during SCN infection (Hewezi et al., 2012). A GRAS family transcription factor (*SCR:* SCARECROW) is predicted as the target of gma-miR9722. In watermelon, the regulatory role of homolog of this gene has been revealed in red light-induced systemic resistance against RKN infection (Lv et al., 2021). In RAD, MSTRG.2711.1-gma-miR5369 and MSTRG.26588.1-gma-miR9725 are introduced as novel potential regulatory components in response to reniform. Auxin influx transporter (*AUX1*; gma-miR5369) is suggested to have a role in the establishment and maintenance of the nematode feeding sites by auxin transporting. Since auxin is essential for syncytia formation, levels of auxin have been found to be increased in nematode-infected roots (Hammes et al., 2005; Ng et al., 2015; Lv et al., 2021). Allene oxide synthase (*AOS*; gma-miR9725 predicted target gene) which is involved in oxylipin JA biosynthesis was detected to be induced by nematode infestation. The regulatory role of this gene in response to nematodes has been functionally investigated. For instance, loss of *AtAOS* gene function in Arabidopsis resulted in improved nematode resistance (Naor et al., 2018). The downregulation of *AOS* gene in infected samples of RAD supports the role of this gene in nematode resistance.

In this study, identified DE-lncRNA and DEG sets were employed to construct lncRNA-mRNA co-expression networks of soybean under SCN and reniform invasion using WGCNA. Under SCN infection, novel relationships were detected between the DE-lncRNAs and neighboring key nematode-associated genes in different modules, which can decipher lncRNA/mRNA interplay-mediated immune response under nematode infection in soybean. For instance, co-expression of DE-lncRNAs with already known nematode-associated DEGs involved in modification of cell wall structure in response to SCN infection, such as *HRGP* and *XTH43*, and signaling cascade genes, such as *LRR-RLKs, CIPK*, and *BAK1*, was found. This was in agreement with previous studies on the involvement of these genes in soybean responses to (Jain et al., 2016; Tang et al., 2017; Zhao et al., 2017; Ma et al., 2020; Niraula et al., 2020). In addition to nematode-responsive protein-coding genes, the identification of co-expressed TFs could reveal the potential involvement of neighboring co-expressed lncRNAs in immune response. In this regard, TFs, such as SCR and TCP4, as well as bZIP and WRKY TF family members, were found with a known protective regulatory role against nematode infection in soybean and other species (Zhao et al., 2015; Neupane et al., 2019a,b; Lv et al., 2021). The potential association between DE-lncRNAs and a RING/U-box superfamily gene, an Aldolase-type TIM barrel family gene, laccase7, *ARF8*, a MYB domain-containing gene, and several *HSP*s, which are involved in resistance to SCN (Zhao et al., 2017; Ul Haq et al., 2019; Cheng et al., 2020; Hishinuma-Silva et al., 2020), brings new insights into the SCN-soybean interaction. Moreover, the association between lncRNAs and nitrite reductase as a major gene in reactive nitrogen species (RNS) metabolism and plant defense against the beet cyst nematode (Labudda et al., 2020), ACC oxidase which is involved in ethylene-based signal transduction and regulates the attractiveness of soybean roots to SCN (Tucker et al., 2010; Hu et al., 2017), galactinol synthase and drought-induced gene as neighboring nematode defense-related protein-coding genes (Kandoth et al., 2011), and *NAC* and *MADS-box* as key TFs involved in soybean defense response under RKN invasion (de Sá et al., 2012) provides important notes for further characterization of SCN resistance genes in soybean. The possible association between DE-lncRNAs and the pectin lyase-like superfamily protein as a pectin-degrading enzyme suggests pectin lyase-like superfamily gene/DE-lncRNA interaction-mediated defense signals *via* modification of cell wall integrity and lignin content, which is consistent with the obtained results in previous studies (Gallego-Giraldo et al., 2020). UDP-D-galactose 4-epimerase5 which confers resistance to the cyst nematode *Heterodera schachtii* (Wubben et al., 2004), *PIP* and *WOX* which turned out to be involved, respectively, in auxin-dependent cell elongation and modulating developmental pathways after pathogen attachment (Maure et al., 2008; Olmo et al., 2020), the *ABC* transporter family gene which participates in the inhibition of hatch and repulsion of potato cyst nematodes (*Globodera pallida* and *G. rostochiensis*) through modulating root composition (Ochola et al., 2021), *EXP*s as cell wall-modifying genes functioning in host defense suppression (Ali et al., 2015; Samalova et al., 2020), and *ANNAT8* and histidine kinase demonstrated to be altered by nematodes for feeding site formation (Dowd et al., 2017; Zhao et al., 2019) were the most important nematode-responsive genes which showed the potential relationship with DE-lncRNAs identified in this study.

Similar to SCN data set, In RAD, the novel associations among DE-lncRNAs and the major nematode-responsive DEGs were elegantly recognized. *NTF2* as overexpressed regulators in infected resistant soybean roots (de Sá et al., 2012), RING/FYVE/PHD zinc finger superfamily protein as positive regulator of downstream targets under nematode infection to establish resistance at transcriptional level (Song et al., 2019), *GSH* introduced in the modulation of giant cell metabolism and manipulation of ROS pathway (Baldacci-Cresp et al., 2012; Wu, 2019), glycosyl hydrolases family with a cell wall-modifying role (Nyaku et al., 2013), Aquaporin-like superfamily gene as a membrane transport protein-coding gene and nematode-induced gene (Hammes et al., 2005), signal peptide peptidase-like2 as a defense response inducer (Harrison et al., 2021), and CAP superfamily gene as highly induced protective gene (Hamamouch et al., 2011) were some nematode response-associated DEGs identified modules of RAD which showed interaction with DE-lncRNAs.

## Conclusion

Nematodes are the most damaging biotic stressors to soybean. Understanding the defense mechanism of soybean plants against nematodes is thus important to take effective biotechnological strategies against them. The role of lncRNAs in the signaling and regulation of diverse plant physiological traits has been revealed in recent years. In the current study, their potential role *via* multiple cis and trans-regulatory mechanisms against SCN and reniform nematodes was predicted in soybean. Over 500 potential lncRNAs were identified in the soybean genome which showed expression under the infection of these two nematode species. Our bioinformatic investigations showed that already known nematode-responsive genes can be cis-targets of these lncRNAs, which are adjacent genes to these sequences in the genome and their expression can be controlled by these >200-nt RNA molecules. Moreover, expression pattern analyses, co-expression studies, and sequence complementation discovered putative nematode-responsive genes among potential trans-targets of soybean lncRNAs. Finally, we identified ~20 lncRNAs as miRNA endogenous target mimics in response against SCN and reniform nematodes. These findings imply an extensive and sophisticated role of lncRNAs in signaling and regulation of biotic responses in plants and can facilitate improving suitable approaches to defeat pathogens in agriculturally important crops. Besides, the potential lncRNAs introduced in this study could further investigate *in planta* to verify their function in response to pathogens.

## Data Availability Statement

Publicly available datasets were analyzed in this study. This data can be found at: NCBI Sequence Read Archive under accession number PRJNA306741 (https://www.ncbi.nlm.nih.gov/bioproject/?term=PRJNA306741) and PRJNA348534 (https://www.ncbi.nlm.nih.gov/bioproject/?term=PRJNA348534).

## Author Contributions

AS, MK, and RK contributed to designing the experiment. AS and MK analyzed and interpreted the data. MK and AS collected samples and extracted RNAs. AS, MK, and SA wrote the paper. All authors contributed to the article and approved the submitted version.

## Conflict of Interest

The authors declare that the research was conducted in the absence of any commercial or financial relationships that could be construed as a potential conflict of interest.

## Publisher’s Note

All claims expressed in this article are solely those of the authors and do not necessarily represent those of their affiliated organizations, or those of the publisher, the editors and the reviewers. Any product that may be evaluated in this article, or claim that may be made by its manufacturer, is not guaranteed or endorsed by the publisher.

## Footnotes

^1^ https://www.statista.com/

^2^ https://github.com/tseemann/barrnap

^3^ http://www.cuilab.cn/lnctar

## Abbreviations

RAD: Reniform-associated data set
dpi: Days post-infection
DAI: Days after inoculation
NFSs: Nematode feeding sites
