# Supplementary material for: Identification of the Complex Interplay Between Nematode-Related lncRNAs and Their Target Genes in Glycine max L
Source: Front Plant Sci. 2021 Dec 10;12:779597. doi: 10.3389/fpls.2021.779597 (PMC8705754; doi:10.3389/fpls.2021.779597)
Supplement: Supplementary file 2 [file Data_Sheet_2.pdf]

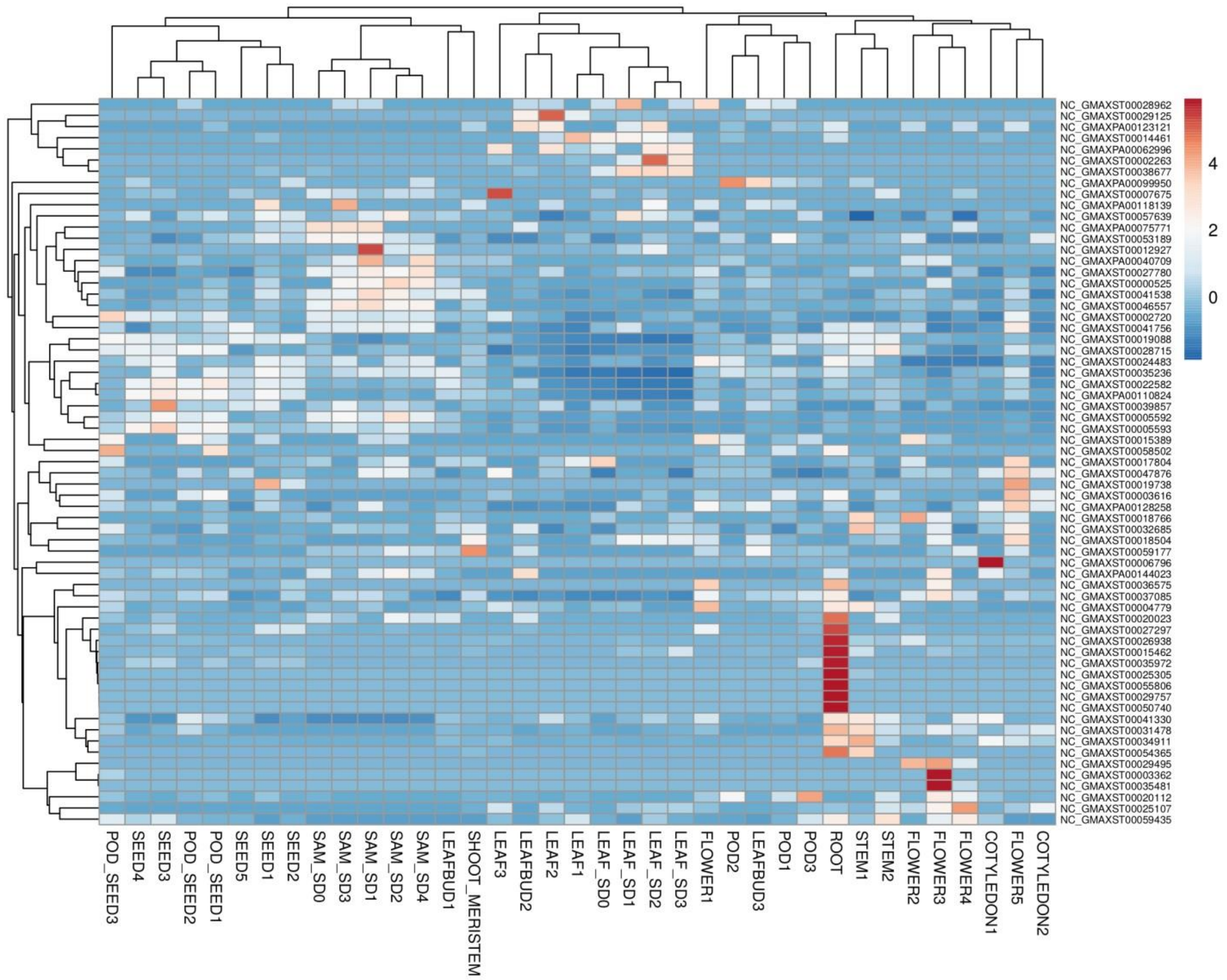

Figure S1. The heatmap showing the expression profile of conserved SCN-lncRNAs that had significant homology with lncRNAs in Golicz's study and were highly expressed in soybean roots. The expression data in different tissues has been provided by Golicz, et al. (2018). The color scale represents the calculated Z-score. The hierarchical clustering of lncRNAs and samples is shown in the dendrogram on the top and side of the heatmap using the complete linkage approach.

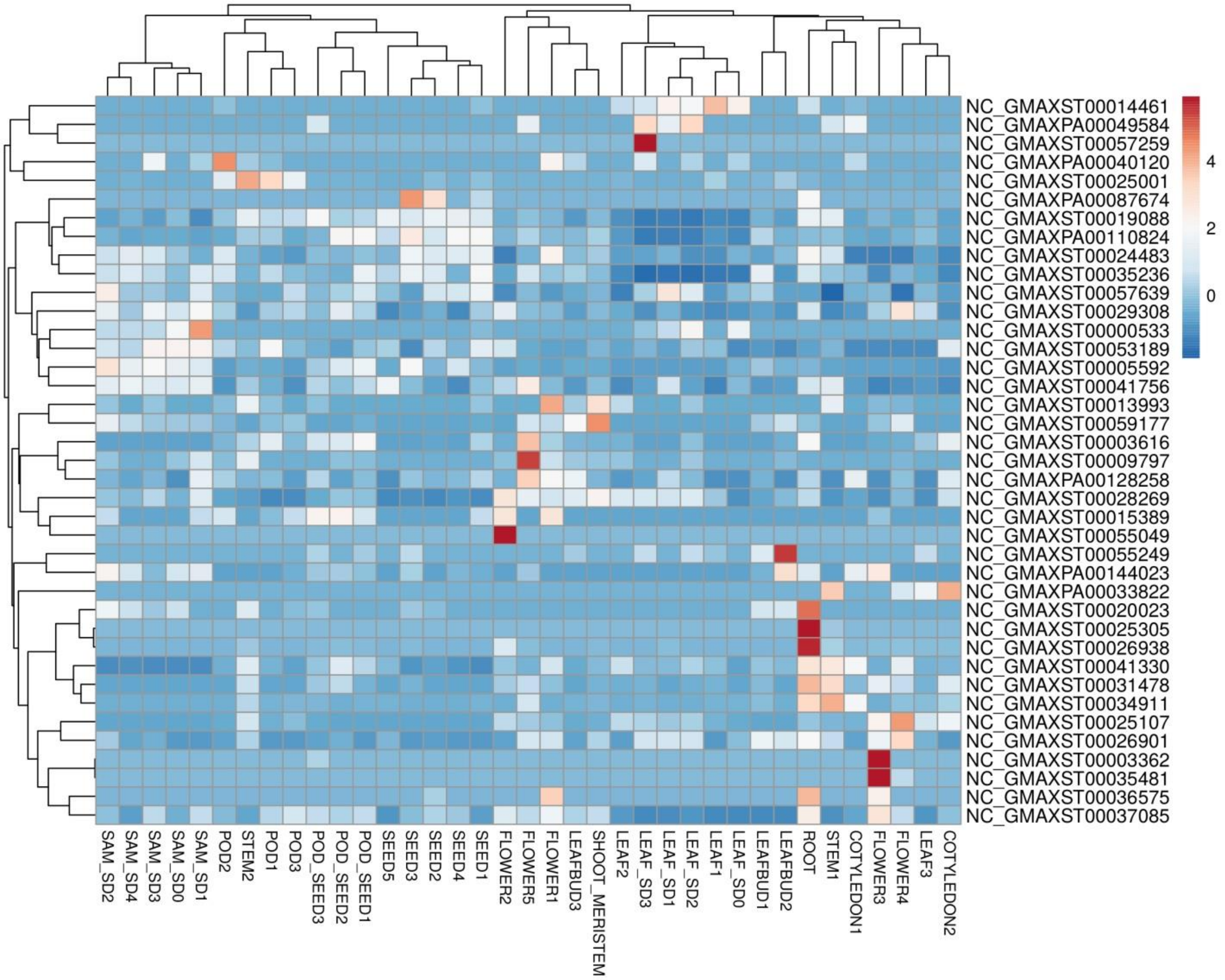

Figure S2. The heatmap showing the expression profile of conserved RAD-lncRNAs that had significant homology with lncRNAs in Golicz's study and were highly expressed in soybean roots. The expression data in different tissues has been provided by Golicz, et al. (2018). The color scale represents the calculated Z-score. The hierarchical clustering of lncRNAs and samples is shown in the dendrogram on the top and side of the heatmap using the complete linkage approach

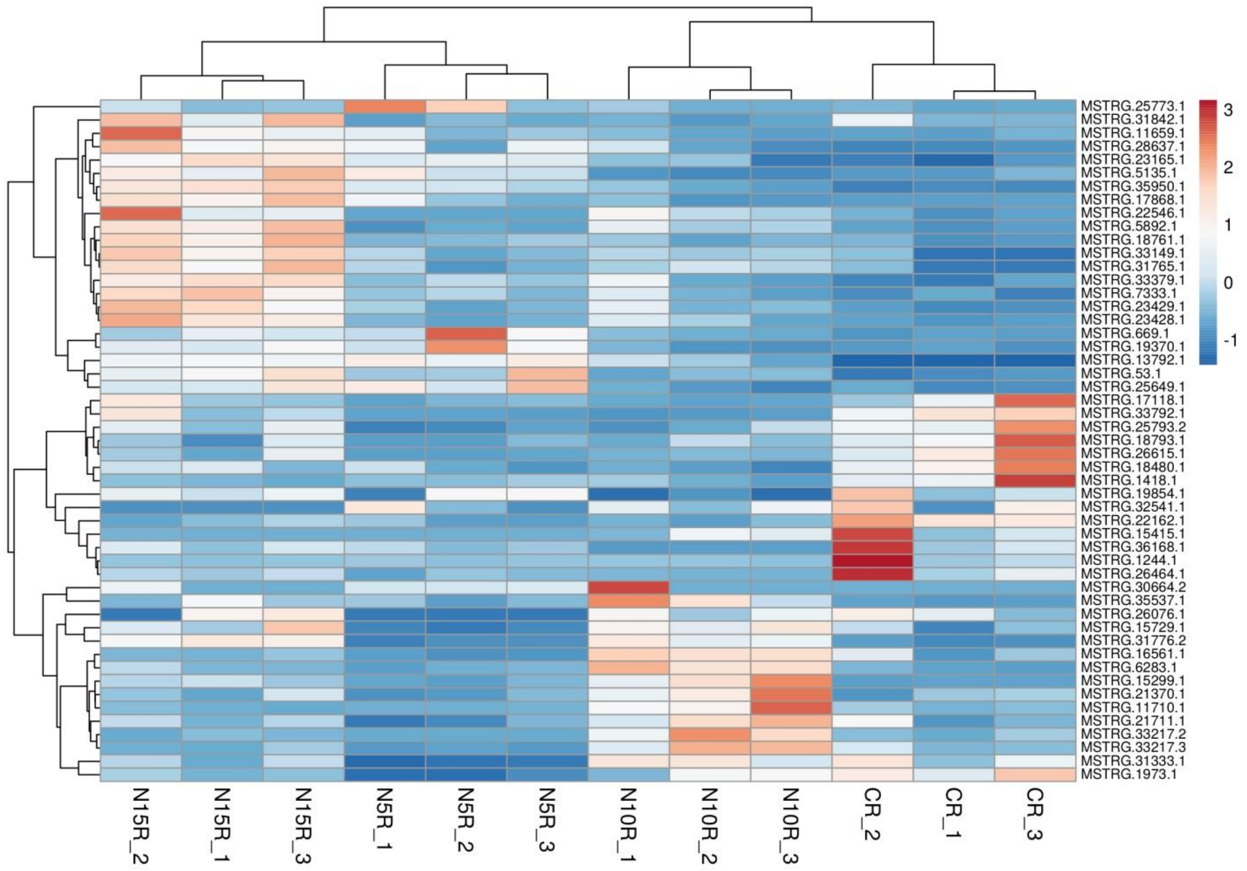

Figure S3. The heatmap of SCN DE-lncRNAs. A correlation analysis using FPKM values indicats patterns of changes in transcript abundance of SCN DE-lncRNAs for each biological replicates in each time-point. The color scale represents the calculated Z-score. The hierarchical clustering of lncRNAs and samples is shown in the dendrogram on the top and side of the heatmap using the complete linkage approach.

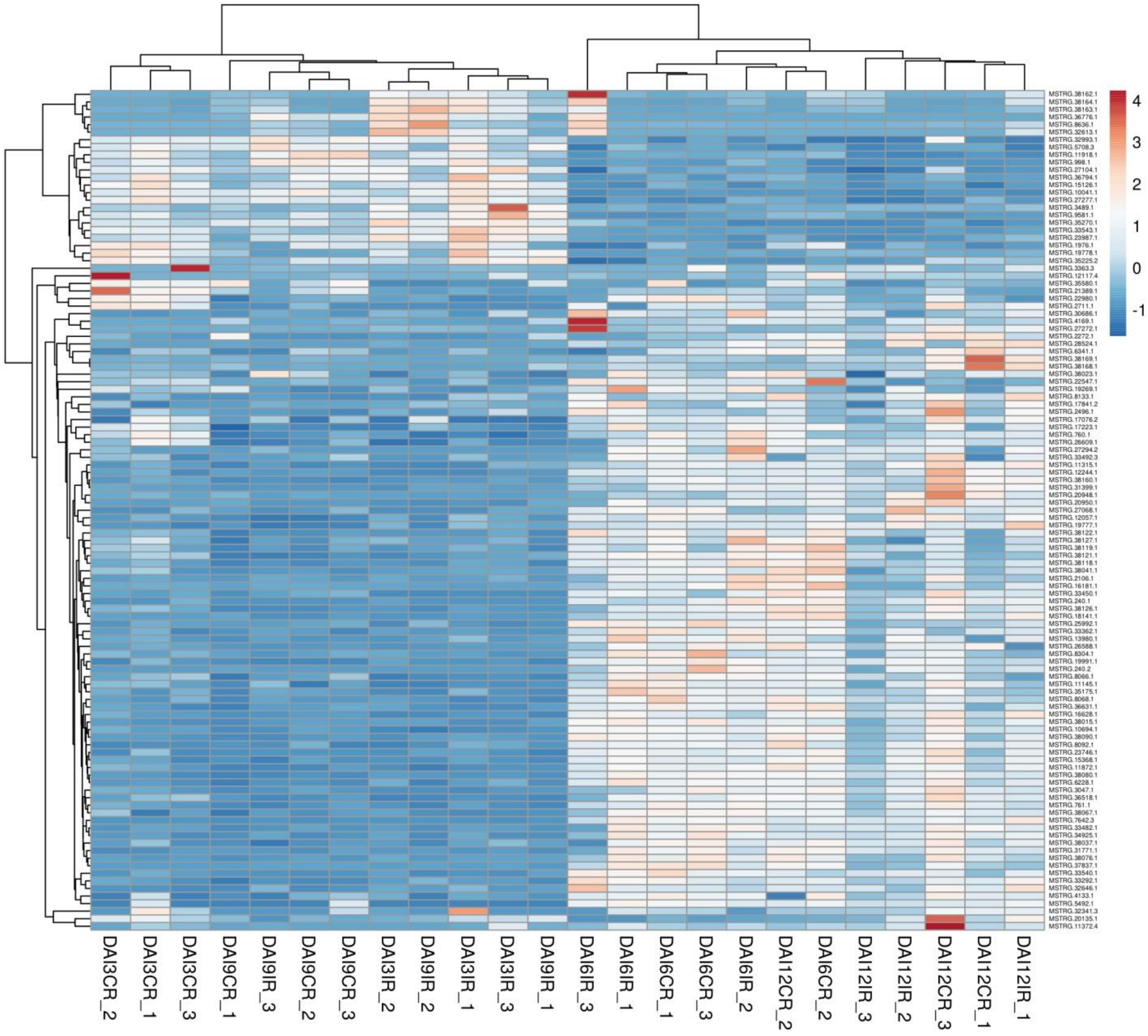

Figure S4. The heatmap of RAD DE-lncRNAs. A correlation analysis using FPKM values indicats patterns of changes in transcript abundance of RAD DE-lncRNAs for each biological replicates in each time-point. The color scale represents the calculated Z-score. The hierarchical clustering of lncRNAs and samples is shown in the dendrogram on the top and side of the heatmap using the complete linkage approach.

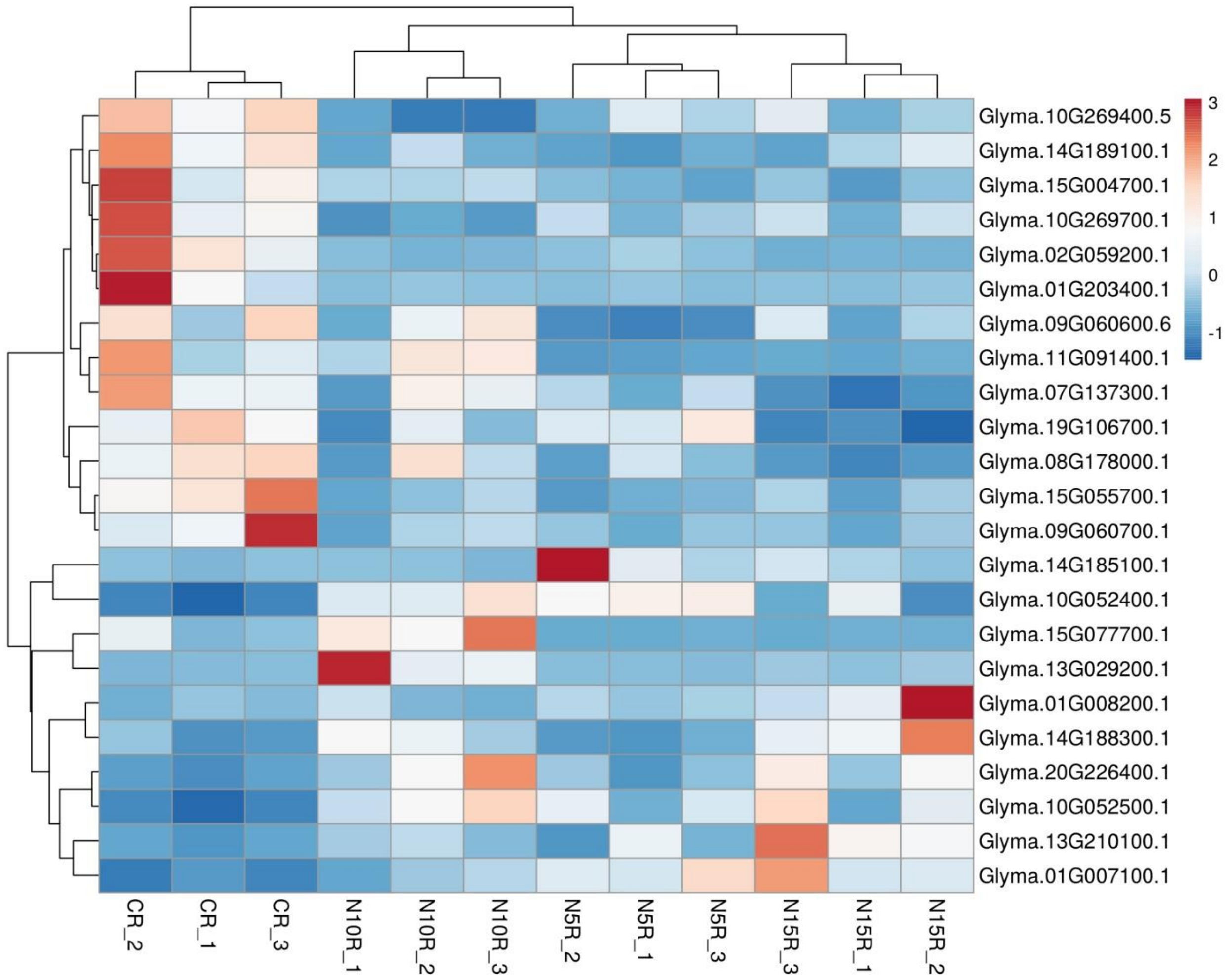

Figure S5. The heatmap showing the expression profile of SCN DE-targets of DE-lncRNAs across all samples. The hierarchical clustering of lncRNAs and samples is shown in the dendrogram on the top and side of the heatmap using the complete linkage approach.

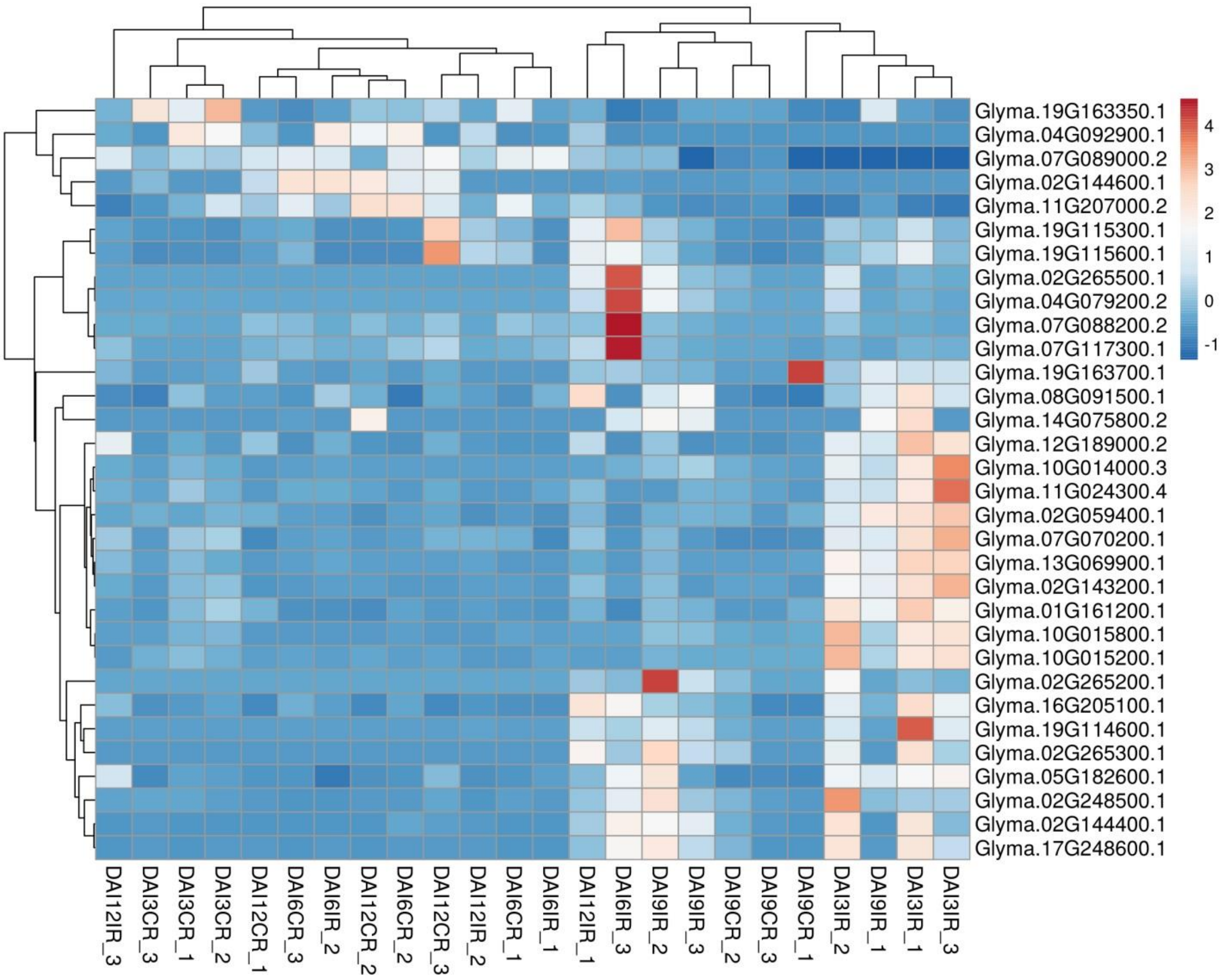

Figure S6. The heatmap showing the expression profile of RAD DE-targets of DE-lncRNAs across all samples. The hierarchical clustering of lncRNAs and samples is shown in the dendrogram on the top and side of the heatmap using the complete linkage approach.

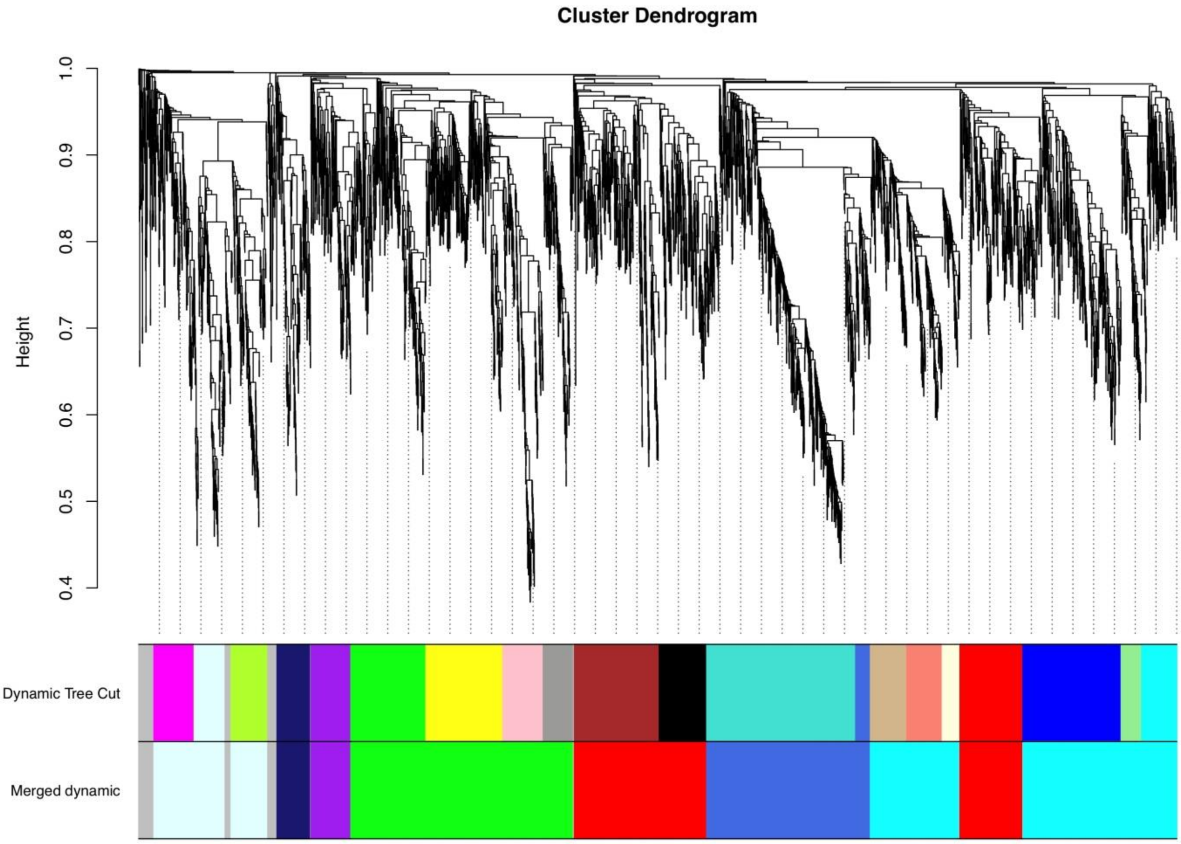

Figure S7. Hierarchical cluster tree displaying the co-expression modules identified in SCN datasset. The branches constitute ten unique modules, and each line represents one gene. Original modules and merged modules are depicted as colored bands below the cluster tree.

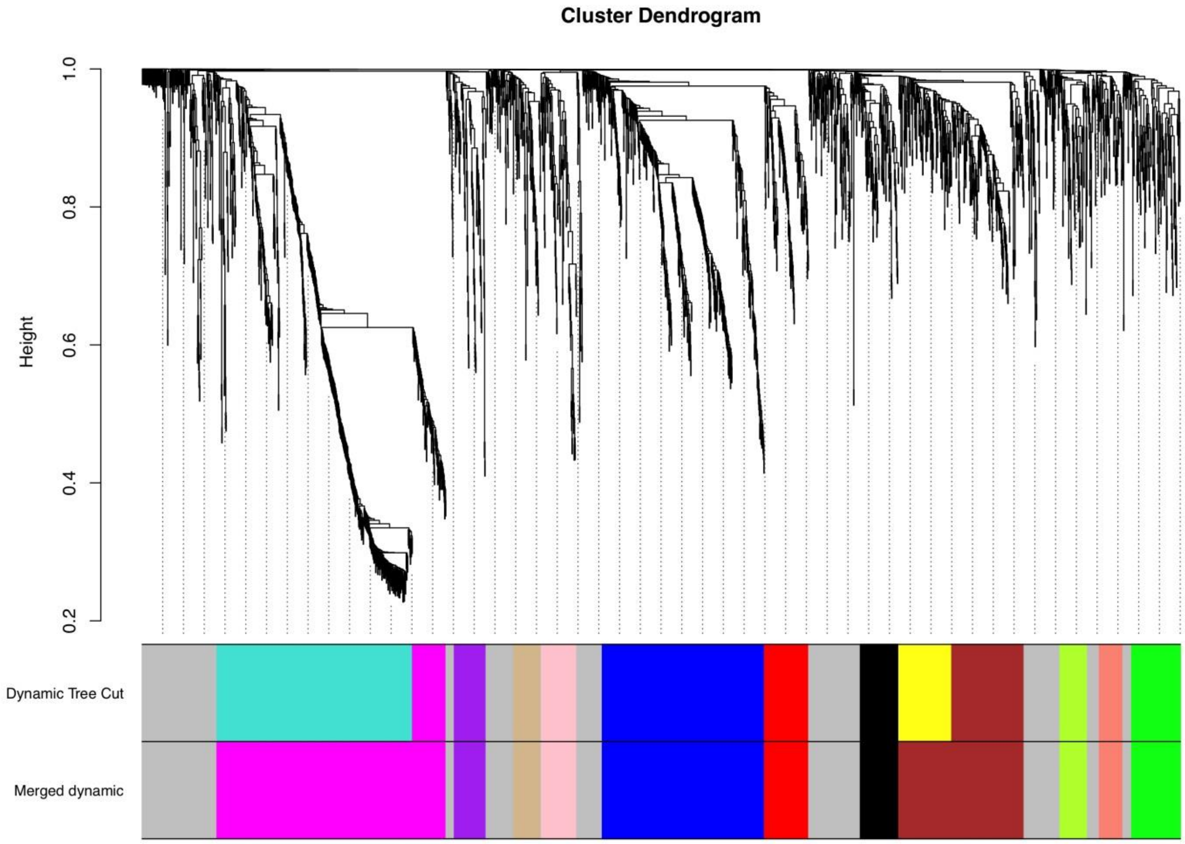

Figure S8. Hierarchical cluster tree displaying the co-expression modules identified in RAD dataset. The branches constitute ten unique modules, and each line represents one gene. Original modules and merged modules are depicted as colored bands below the cluster tree.
